# Evaluating the use of rodents as in vitro, in vivo and ex vivo experimental models for the assessment of tyrosine kinase inhibitor-induced cardiotoxicity: a systematic review

**DOI:** 10.1007/s00204-025-04159-0

**Published:** 2025-09-11

**Authors:** Lucy Gill, Amy Chadwick, Annette P. Meeson, Richard Barrett-Jolley, Marie M. Phelan, Rachel A. Oldershaw

**Affiliations:** 1 Department of Musculoskeletal and Ageing Science, Institute of Life Course and Medical Sciences, Faculty of Health and Life Sciences, William Henry Duncan Building, 6 West Derby Street, Liverpool, L7 8TX UK; 2 Centre for Drug Safety Science, Department of Pharmacology & Therapeutics, Institute of Systems, Molecular and Integrative Biology, Faculty of Health and Life Sciences, University of Liverpool, Sherrington Building, Ashton Street, Liverpool, L69 3GE UK; 3 Biosciences Institute, Faculty of Medical Sciences, Newcastle University, International Centre for Life, Central Parkway, Newcastle Upon Tyne, NE1 3BZ UK; 4 Department of Biochemistry, Institute of Systems, Molecular and Integrative Biology, Faculty of Health and Life Sciences, University of Liverpool, Biosciences Building, Crown Street, Liverpool, L7 7BE UK; 5 High Field NMR Facility, Liverpool Shared Research Facilities (LIV-SRF), Faculty of Health and Life Sciences, University of Liverpool, Crown Street, Liverpool, L69 7ZB UK

**Keywords:** Drug-induced cardiotoxicity, Tyrosine kinase inhibitor, Systematic review, Rodent models, Preclinical models, Risk of bias analysis, Drug discovery

## Abstract

Tyrosine kinase inhibitors (TKIs) are widely used in cancer therapy yet are strongly associated with acute and chronic cardiotoxicity in patients. There is a critical need to advance our understanding of the pathophysiology that underlies TKI-mediated cardiotoxicity, and central to this is the use of reproducible and relevant preclinical models, which are employed in the evaluation of TKIs across the drug discovery pipeline. We have conducted a systematic review of the literature to determine how rodent models are used in the measurement of TKI-induced cardiotoxicity, focusing on animal reports, physiological cardiac outputs, histopathology, and biomarkers. A PRISMA-compliant systematic review was conducted using PubMed, Scopus, and Web of Science to identify studies reporting on TKI-induced cardiotoxicity in rodents. Only controlled in vivo, primary in vitro, and ex vivo studies using rats, mice, hamsters or guinea pigs were included. Data were extracted on species, strain, sex, age, experimental design, and cardiac outcomes with risk of bias analyses performed using the SYRCLE and SciRAP tools. Among 92 studies, sunitinib, imatinib, and sorafenib were the most frequently examined TKIs, with cardiotoxicity exhibited as altered cardiac functional parameters, fibrotic changes, arrhythmias, and elevated cardiac biomarkers. Rats (51 studies) and mice (46 studies) were predominantly used to study the effects of TKIs, whilst guinea pigs were underrepresented, limiting insights into electrophysiological changes that are associated with cardiotoxicity. Most studies used male rodents, and only two studies assessed age-related effects. Comparison between species strains was rarely conducted, despite evidence of this being a contributing factor to pre-disposition to cardiotoxicity. Rodent models were shown to replicate TKI-induced cardiotoxic effects observed in humans, but risk of bias analyses revealed limited evidence for study randomisation, inconsistent blinding, lack of sex-balanced studies, and poor strain diversity. Poor methodological quality and reporting across studies compromised reproducibility and interpretation of clinical relevance. Our study highlights the need for implementation of standardised protocols, strain, sex and age-stratified analyses to better support preclinical-to-clinical translation, as well as improve the safety of TKIs for patients and ensure more ethical use of animals in research.

**Supplementary Information:**

The online version contains supplementary material available at 10.1007/s00204-025-04159-0.

## Introduction

Cardiotoxicity is an adverse side effect of many cancer therapies, which manifests as myocardial dysfunction, cardiomyopathies, arrhythmias, heart failures and sudden cardiac death (Sayegh et al. 2023). In addition to affecting the quality of life of cancer patients, cardiotoxic events can lead to premature cessation or interruption of treatment, reducing therapeutic efficacy (Porter et al. 2022). Cardiotoxicity can occur both acutely, at the beginning of treatment, or chronically up to years after end of treatment (Kaddoura et al. 2023). Moreover, clinical identification of cardiotoxicity can be difficult to predict and requires long-term cardiac monitoring before, during and after treatment (Shyam Sunder et al. 2023).

**Fig. 1:**
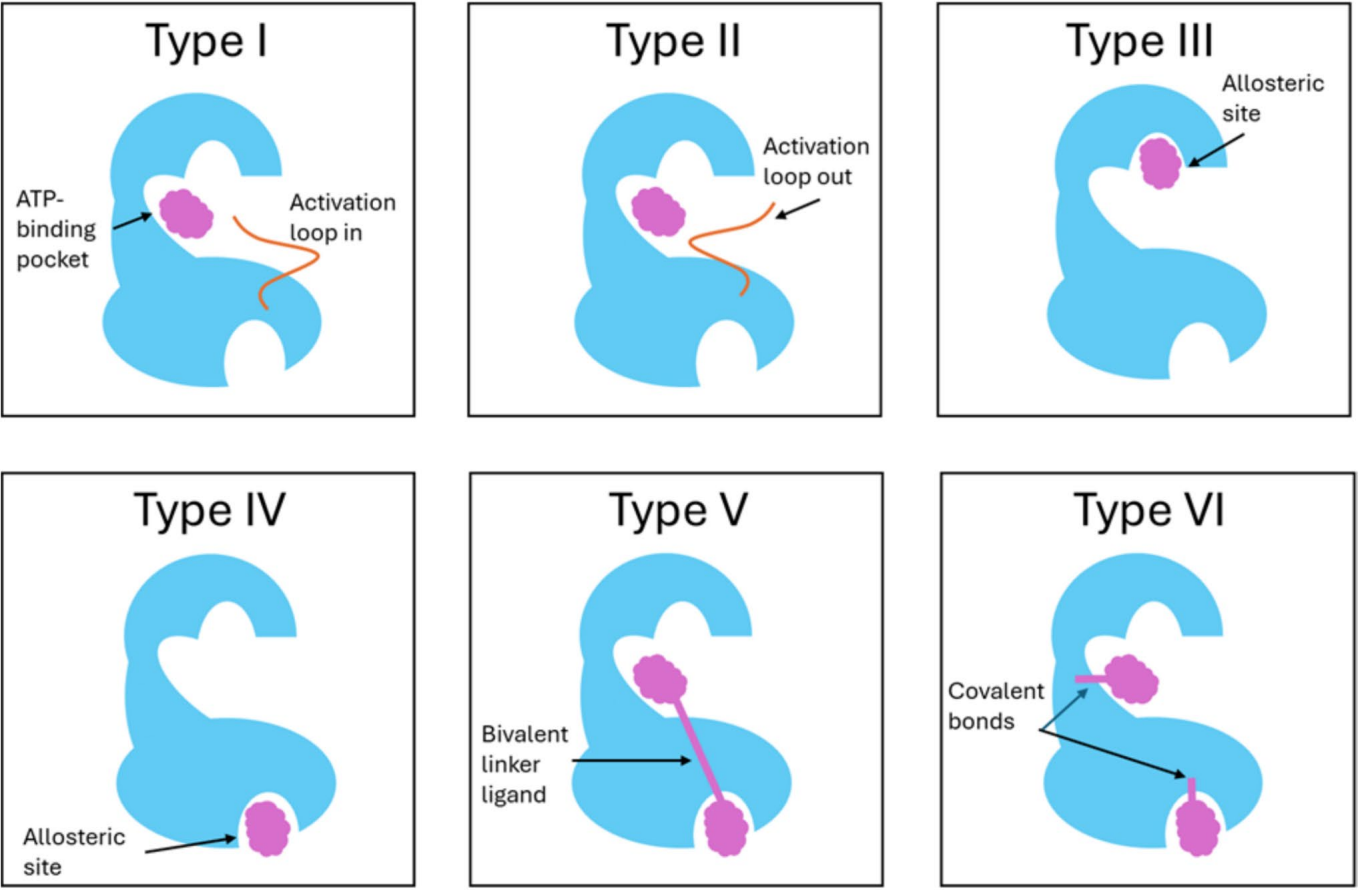
Classification of Tyrosine Kinase Inhibition. The six mechanisms of tyrosine kinase inhibition based on their binding site and mode of interaction. Type I and II inhibitors bind the ATP pocket, stabilising active and inactive conformations, respectively. Type III targets an allosteric site within the ATP binding domain whilst Type IV binds to an allosteric site away from the ATP binding domain. Type V inhibitors are bivalent, binding both ATP and allosteric sites. Type VI inhibitors form covalent bonds with the kinase. Adapted from Martinez et al (2020) Part A: Type III Kinase Inhibitors (Martinez et al. 2020)

Tyrosine kinase inhibitors (TKIs) have revolutionised cancer therapy by blocking the phosphorylation of tyrosine residues within the key signalling pathways that regulate tumour growth, survival, and angiogenesis. The exact mechanism of kinase inhibition varies between TKIs, with six known modes based on the interaction with the ATP-binding pocket and adjacent regulatory regions (Fig. 1) (Kumar et al. 2024; Martinez et al. 2020; Wu et al. 2015). Commonly targeted tyrosine kinases include BCR-ABL, a fusion protein found in chronic myeloid leukaemia (Joensuu and Dimitrijevic 2001; Reddy and Aggarwal 2012), epidermal growth factor receptor (EGFR), which is frequently mutated in lung cancers promoting cellular proliferation or activation of anti-apoptotic pathways (Bethune et al. 2010), and vascular endothelial growth factor receptors (VEGFR), essential for tumour angiogenesis (Huang et al. 2020; Patel et al. 2023).

Since the development and approval of imatinib in 2001, research into TKIs have come to the forefront of cancer therapy research (Joensuu and Dimitrijevic 2001). Over 50 TKI’s have been approved worldwide for clinical use in humans as cancer therapeutics and some autoimmune disorders (MRC Protein Phosphorylation and Ubiquitylation Unit. 2025). Despite their success, there are growing concerns over their effects on the cardiovascular system.

Given the expanding position of TKIs within the drug development pipeline, it is essential to improve our understanding and detection of TKI-induced cardiotoxicity. The proposed mechanisms underlying TKI-induced cardiotoxicity are varied. Some TKI’s such as nilotinib and vandetanib, have been found to induce arrythmias such as QT prolongation, which is related to the inhibition or downregulation of key potassium channels such as HERG (Cui et al. 2022). The induction of mitochondrial dysfunction in cardiomyocytes has also been proposed, with sorafenib impairing mitochondrial function at clinically relevant concentrations (Will et al. 2008). It has also been suggested that ‘bystander’ kinases, which are not essential for tumour cells but, are involved in cardiomyocyte survival may be inhibited ‘off-target’ as many TKIs are multi-targeted (Force et al. 2007). Additionally, the inhibition of VEGFR signalling by many TKIs has been linked to the development of hypertension (Robinson et al. 2010), which can contribute to the development of heart failure (Oh and Cho 2020).

Rodent models are widely used in pharmaceutical research and preclinical safety assessment to investigate drug-induced cardiotoxicity (Kettenhofen and Bohlen 2008). There are economic benefits to their use due to their small size, housing requirements, large litters and rapid development (Bryda 2013). In addition to these financial benefits, there are clear biological advantages such as genetic similarity, with mice sharing 99% of genes with humans. This similarity facilitates the creation of genetically modified rodents that can be used as developmental and disease models that replicate the human condition (Waterston et al. 2002). Furthermore, rodents replicate the biological processes observed in human cardiotoxicity, including cardiac remodelling, fibrosis, mitochondrial dysfunction, and oxidative stress responses, making them valuable tools for studying the molecular mechanisms underlying TKI-induced cardiac injury (Russell et al. 2005; Wang et al. 2022). Due to their similarity in anatomy, measurement of functional parameters can be performed in both humans and rodents including, echocardiograms, electrocardiograms, blood pressure monitoring and analogous cardiac biomarkers (Farraj et al. 2011; Harrison et al. 2024; Kim et al. 2016; Liu and Rigel 2009).

The predictive value of rodent models in evaluating drug-induced cardiotoxicity is however limited by several factors. There are key differences between rodents and humans, including faster heart rates and differing ion channel expression profiles, which in turn leads to distinct ECG patterns both between humans and rodents and between rodent species (Supplemental Figure 1). In humans, ventricular repolarisation predominantly relies on IKr and IKs currents, which have minimal contribution to cardiomyocyte electrophysiology in rats and mice (Li et al. 1996) (Joukar 2021). Instead, rats and mice rely on Ito and IKur, which create faster and sharper repolarisation and lack a plateau phase in the ventricular action potential (Boukens et al. 2014; Li et al. 2004). Guinea pigs lack Ito and depend more on IKs but lack phase 1 (Gussak et al. 2000; Sanguinetti and Jurkiewicz 1991). Rat and mice ECGs typically feature a prominent J wave, shortened QT intervals, absent Q waves, and poor separation between QRS and T waves, making interpretation of QT prolongation and related disturbances challenging (Gussak et al. 2000; Joukar 2021). These differences could limit the predictive value of rodent models in evaluating TKI-induced pro-arrhythmic risk.

### Rationale for a systematic review of rodent models used to investigate TKI-induced cardiotoxicity

Despite widespread use of rodent models in preclinical cardiotoxicity research, there is currently no comprehensive synthesis, which evaluates how effective these models are at capturing the full spectrum of TKI-induced cardiotoxicity. Study design, species selection, dosing regimens, and the choice of outcome metrics vary widely between experiments, making it difficult to draw overarching conclusions about the consistency and translational relevance of these models.

Therefore, a systematic review is necessary to map and critically evaluate the methodologies and findings from existing rodent studies investigating TKI cardiotoxicity. By identifying patterns, inconsistencies, and potential gaps in the evidence, this review will help determine whether rodent models reliably capture TKI-induced cardiac risks and identify where optimisation may be needed to improve their predictive value for human cardiotoxicity. Furthermore, by highlighting opportunities for standardisation and identifying areas where unnecessary duplication of studies could be avoided, the review can contribute to the principles of Replacement, Reduction, and Refinement (3Rs), ultimately promoting more ethical and efficient use of animals in cardiotoxicity research (Russell and Burch 1959).

We hypothesised that rodent models exhibit TKI-induced cardiotoxicity, with similar clinical observations in human patients. The main objective was to conduct a PRISMA-compliant systematic review to synthesise and critically assess available evidence regarding the use of rodent models in cardiotoxicity studies with TKIs. Outcomes of TKI-induced cardiotoxicity were evaluated with three groups considered based on study type: (1) in vivo experiments, defined as TKI administered to live rodents, (2) in vitro experiments, defined as primary cardiomyocytes, isolated from untreated rodents, which were cultured and then exposed to a TKI and (3) ex vivo, defined as where intact cardiac tissues or organs were excised from untreated rodents and then exposed to a TKI under controlled laboratory conditions.

## Methods

### PROSPERO registration and PRISMA protocol

The study was prospectively registered on PROSPERO a priori (CRD42021231446). The Preferred Reporting Items for Systematic Review and Meta-Analyses (PRISMA) protocol (Supplemental Table 1, Moher et al. 2009; Page et al. 2021)) was established to define the ‘Population, Intervention, Comparison, Outcomes and Study design’ (PICOS) framework with inclusion of the aims and objectives of the study, bibliographic database, search terms, and inclusion/exclusion criteria (Supplemental Table 2, Amir-Behghadami and Janati 2020).

### Search strategy

A comprehensive search was conducted for relevant studies without restriction using bibliographic databases PubMed, Web of Science, and Scopus with the last date of access being June 2025. The search terms included a combination of keywords related to rodent models, tyrosine kinase inhibitors and cardiotoxicity outcomes (Supplemental Table 3). No other systematic reviews on TKI-induced cardiotoxicity evaluation in rodent species were identified for screening and inclusion of additional studies.

### Study selection and identification of eligible studies

**Fig. 2:**
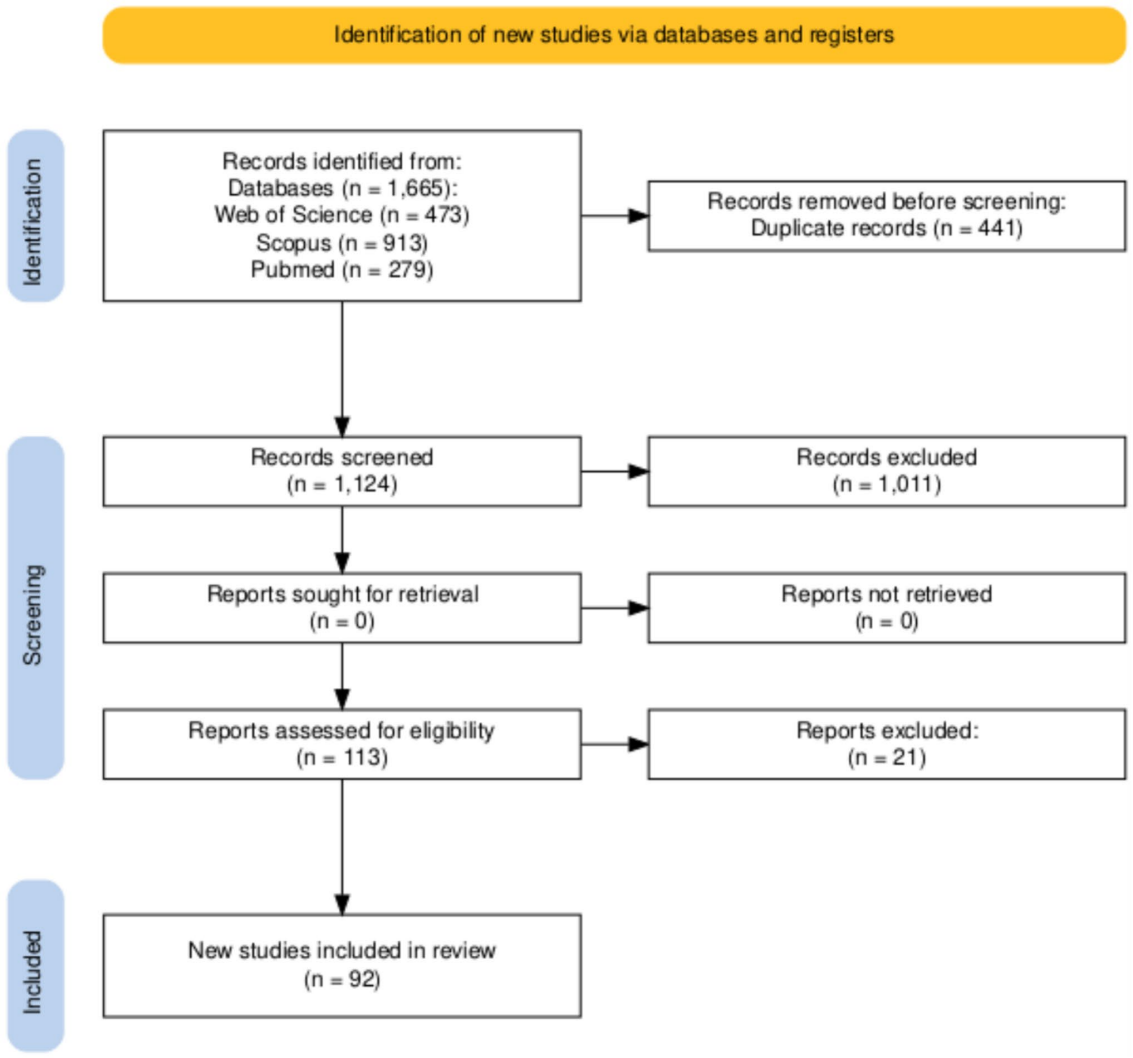
PRISMA Flow Diagram Detailing the Systematic Review Selection Process. Studies retrieved from bibliographic databases were systematically screened against the inclusion and exclusion criteria. Arrows directed downwards represent sequential steps in screening for study eligibility, whereas arrows directed to the right indicate the numbers of studies excluded at each stage

Studies returned from bibliographic database searches were collated in EndNote reference manager and duplicate study entries removed. Retrieved studies were screened by following the PRISMA flow diagram, created using the PRISMA2020 Shiny app (https://estech.shinyapps.io/prisma_flowdiagram/) and checklist (Fig. 2 and Supplemental Table 1) against the inclusion and exclusion criteria defined in Supplemental Table 4. Search results were uploaded to the Rayyan QCRI web application (https://rayyan.qcri.org/cite) (Ouzzani et al. 2016) and screened by title and abstract to exclude studies that were indisputably irrelevant. Further in-depth scrutiny of the full text of remaining studies was performed independently by LG, MMP, AC, RBJ and RAO followed by discussion and consensus used to resolve conflicts.

### Quality assessment and risk of bias assessments

Standardisation was performed by risk of bias analysis of the reporting quality for each study. The SYRCLE’s risk of bias tool was applied to in vivo studies (Hooijmans et al. 2014), with further adaptation for ex vivo studies (Supplemental Table 5). For in vitro studies, the SciRAP risk of bias tool was used (Supplemental Tables 6 and 7, (Roth et al. 2021)).

### Data extraction

Data was extracted into Excel spreadsheets according to the PICOS framework (Supplemental Table 2) to include study design, rodent model characteristics, details of the TKIs studied, and significant cardiac outcomes.

### Data analysis

A narrative synthesis was performed by grouping studies according to the type of TKI, rodent species, and the format of the experimental model: in vivo (TKI administered to live rodents), ex vivo (cardiac tissues or organs excised from untreated rodents and then exposed to a TKI), or in vitro (primary cardiac cells isolated from untreated rodents and subsequently exposed to a TKI). Continuous data, such as ejection fraction and biomarker levels, were analysed to identify inter-study associations and variability. Histopathological and clinical outcomes were summarised to provide insights into the cardiotoxic effects of TKIs across different rodent models.

## Results

### Study selection

A total of 1665 records were retrieved from three bibliographic databases: Web of Science 473, Scopus 913, and PubMed 279. No other systematic reviews relating to the evaluation of TKI-induced cardiotoxicity in rodent models were found for inclusion of additional studies. Following removal of 441 duplicate records, a total of 1224 studies were screened by title and abstract against the inclusion and exclusion criteria to leave 113 studies remaining for potential inclusion. Consensus examination of each remaining article excluded a further 21 studies to produce a final dataset of 92 studies for data extraction and analysis (Table 1 and Supplemental Table 8). The outputs of the study selection process are summarised in the PRISMA flow diagram (Fig. 2) with justification for exclusion of studies provided in Supplemental Table 8.

**Table 1:** Study characteristics of rodent species and experimental approaches in TKI-induced cardiotoxicity studies

Study	Year	TKI tested	Rodent species	Strain	Reported rodent numbers	Study type		
						**In vivo**	**Ex vivo**	**In vitro**
Abdelgalil et al. (2020)	2020	SOR	R	W	18	*Y*	**N**	**N**
Abdel-Wahab et al. (2025)	2025	IM	R	W	24	*Y*	**N**	**N**
Aguirre et al. (2010)	2010	SUN	R	SD	114	*Y*	**N**	**N**
Akman et al. (2014)	2014	PZP	R	SD	24	*Y*	**N**	**N**
Alanazi et al. (2022)	2022	GEF	R	W-Albino	32	*Y*	**N**	**N**
AlAsmari et al. (2020)	2020	GEF	R	W-Albino	40	*Y*	**N**	**N**
Aldemir et al. (2020)	2020	SUN	R	W-Albino	30	*Y*	**N**	**N**
Alhazzani et al. (2024)	2024	DAS	R	SD	24	*Y*	**N**	**N**
Alhoshani et al. (2020)	2020	GEF	R	SD	12	*Y*	**N**	**N**
Barr et al. (2014)	2014	IM	R	SD	NR	**N**	**N**	*Y*
Blanca et al. (2016)	2016	SUN	R	W	24	*Y*	**N**	**N**
Blasi et al. (2012)	2012	SUN	R (In vivo) and GP (Ex vivo)	DH GP and SD rat	40 R and NR for GP	*Y*	*Y*	**N**
Bordun et al. (2015)	2015	BEV SUN	M	C57Bl/6	75	*Y*	**N**	**N**
Bouitbir et al. (2019)	2019	SUN	M	C57Bl/6Rj	20	*Y*	**N**	**N**
Burke et al. (2019)	2019	IM SUN	R	W	NR	**N**	**N**	**Y**
Cheng et al. (2023)	2023	CRI	M	C57BL/6	36	*Y*	**N**	**N**
Chintalgattu et al. (2013)	2013	SUN	M	NR	NR	*Y*	**N**	**N**
Cooper et al. (2019)	2018	SUN	R	SD	79	**N**	*Y*	**N**
Cosgun et al. (2021)	2021	VAN	R	W-Albino	18	*Y*	**N**	**N**
Duran et al. (2014)	2014	SOR	M	NR	71	*Y*	**N**	**N**
French et al. (2010)	2010	SUN SOR PZP	R	SD	41	*Y*	**N**	*Y*
Harvey and Leinwand (2015)	2015	SUN	R and M	NR- mice and SD rat	NR	*Y*	**N**	**N**
Hasinoff et al. (2017)	2017	BOS DAS IM NIL PON	R	SD	NR	**N**	**N**	*Y*
Henderson et al. (2013)	2013	SUN SOR ERL	R	SD	45	**N**	*Y*	**N**
Herman et al. (2011)	2011	IM	R	SHR and SD	45 SHR and 25 SD	*Y*	**N**	*Y*
Herman et al. (2014)	2014	IM	R	SD	20	*Y*	**N**	**N**
Heyen et al. (2013)	2013	IM BOS	R	SD	29	*Y*	**N**	**N**
Imam et al. (2020)	2020	SUN	R	W	30	*Y*	**N**	**N**
Jacob et al. (2016)	2016	GEF LAP SUN IM SOR VAN LST	R	W and L	NR	**N**	**N**	*Y*
Jensen et al. (2017a)	2017	SOR	M	FVB/N	20	*Y*	**N**	**N**
Jensen et al. (2017b)	2017	SUN ERL	M	FVB/N	30	*Y*	**N**	**N**
Jiang et al. (2019)	2019	IBR	M	C57BL/6	NR	*Y*	**N**	**N**
Jie et al. (2021)	2021	GEF	GP	DH	NR	**N**	*Y*	**N**
Kerkelä et al. (2006)	2006	IM	M	C57BL/6	NR	*Y*	**N**	**N**
Kobara et al. (2021)	2021	IM	M and R	C57BL/6 M and W	NR	*Y*	**N**	*Y*
Korashy et al. (2016)	2016	GEF	R	W	27	*Y*	**N**	**N**
Krüger et al. (2025)	2025	LEN	M	C57BL/6 J	NR	*Y*	**N**	**N**
Kuburas et al. (2022)	2022	SUN	R	SD	92	**N**	*Y*	**N**
Latifi et al. (2019)	2019	PON DAS	M	C57BL/6	NR	*Y*	**N**	**N**
Li et al. (2019)	2019	SUN	M	C57BL/6	NR	*Y*	**N**	**N**
Li et al. (2022)	2022	SOR	M	C57BL/6 mice	36	*Y*	**N**	**N**
Li et al. (2023a)	2023	SUN	M and R	C57BL/6	NR	*Y*	**N**	*Y*
Li et al. (2023b)	2023	OSM	GP and R	DH GP and SD rats	NR	**N**	*Y*	**N**
Li et al. (2024c)	2024	SOR	M	C57BL/6	18	*Y*	**N**	**N**
Li et al. (2024b)	2024	IBR	M	C57BL/6 J	NR	*Y*	**N**	**N**
Li et al. (2024a)	2024	SUN	M	C57BL/6 J	NR	*Y*	**N**	**N**
Li et al. (2025)	2025	IBR	M	C57BL/6 J	NR	*Y*	**N**	**N**
Lim et al. (2010)	2010	SUN	M	ICR	72	*Y*	**N**	**N**
Liu et al. (2023)	2023	SOR	R	SD	NR	*Y*	**N**	*Y*
Liu et al. (2024)	2024	SOR	M	C57BL/6	24	*Y*	**N**	**N**
Liu et al. (2025)	2025	ALE	R	SD	NR	*Y*	Y	**N**
Ma et al. (2020a)	2020	SOR	R	SD	NR	*Y*	*Y*	*Y*
Maayah et al. (2014)	2014	SUN	R	W-Albino	48	*Y*	**N**	**N**
Madonna et al. (2021)	2021	PON	M	C57BL/6	36	*Y*	**N**	**N**
Madonna et al. (2022)	2022	PON	M	C57BL/6	36	*Y*	**N**	**N**
Maharsy et al. (2014)	2014	IM	M and R	Gata4 ± and Bcl-2-overexpressing mice NR rats	NR	*Y*	**N**	**N**
Mak et al. (2015)	2015	ERL	R	SD	22	*Y*	**N**	**N**
Mao et al. (2012)	2012	SIM010603 SUN	R	SD	100	*Y*	**N**	**N**
Marslin et al. (2015)	2015	IM	R	W	18	*Y*	**N**	**N**
Mattii et al. (2024)	2024	PON	M	C57BL/6	32	*Y*	**N**	**N**
McMullen et al. (2021)	2021	SUN IM	R	SD	NR	**N**	**N**	*Y*
Mohamad et al. (2024)	2024	SUN	R	W-Albino	30	*Y*	**N**	**N**
Monogiou Belik et al. (2024)	2024	QZ	M and R	C57BL/6NRJ- mice and SD rat	153 mice and NR rat	*Y*	**N**	*Y*
Mooney et al. (2015)	2015	SUN	GP	DH	50	*Y*	**N**	**N**
Mozolevska et al. (2019)	2019	BEV SUN	M	C57BL/6	194	*Y*	**N**	**N**
Patyna et al. (2008)	2008	SUN	R	SD	NR	*Y*	**N**	**N**
Pentassuglia et al. (2009)	2009	PKI166	R	W	NR	**N**	**N**	*Y*
Qin et al. (2024)	2024	SUN	M	C57BL/6	40	*Y*	**N**	**N**
Qu et al. (2025)	2025	SUN	M	C57BL/6 J	NR	*Y*	**N**	**N**
Ren et al. (2021)	2021	SUN	M	C57BL/6	40	*Y*	**N**	**N**
Sandhu et al. (2017)	2017	SUN	R	SD	NR	**N**	*Y*	**N**
Savi et al. (2018)	2018	IM	R	W	38	*Y*	**N**	**N**
Sayed-Ahmed et al. (2019)	2019	SUN	R	W-Albino	40	*Y*	**N**	**N**
Shi et al. (2025)	2025	IBR	R	W	NR	*Y*	**N**	*Y*
Shuai et al. (2023)	2023	IBR	R	SD	88	*Y*	**N**	**N**
Song et al. (2022)	2022	IM	M	C57BL/6	26	*Y*	**N**	**N**
Song et al. (2023)	2023	IM	M	C57BL/6 J	36	*Y*	**N**	**N**
Sourdon et al. (2021)	2021	SUN	M	C57BL/6 and NMR nude	23 NMR nude and 28 C57BL/6	*Y*	**N**	**N**
Stuhlmiller et al. (2017)	2017	ERL SUN SOR	M (in vivo) and R (in vitro)	FVB mouse and SD rat	33 mice NR rats	*Y*	**N**	*Y*
Tousif et al. (2023)	2023	PON	M	C57BL/6 J	NR	*Y*	**N**	*Y*
Tuomi et al. (2018)	2018	IBR	M	C57BL/6	NR	*Y*	**N**	**N**
Tuomi et al. (2021)	2021	IBR ACA	M	C57BL/6	NR	*Y*	**N**	**N**
Wolf et al. (2010)	2010	IM	R (In vitro*/vivo*) and M (In vivo)	C57BL/6 mice and SD rat	NR	*Y*	**N**	*Y*
Wolf et al. (2011)	2011	NIL	R (In vitro/*vivo*)	HanRcc:W and SD rat	57 mice NR rat	*Y*	**N**	*Y*
Xiao et al. (2020)	2020	IBR	M	Several strains with different mutations	NR	*Y*	**N**	**N**
Xu et al. (2022)	2022	SUN	M	C57BL/6	NR	*Y*	**N**	*Y*
Xu et al. (2024a)	2024	CRI	M	C57BL/6 J	NR	*Y*	**N**	*Y*
Xu et al. (2024b)	2024	REG	M	C57BL/6 J	NR	*Y*	**N**	**N**
Yan et al. (2024)	2024	IBR	R	SD	70	*Y*	**N**	**N**
Yang et al. (2019a)	2019	SUN	M	NR	129	*Y*	**N**	**N**
Yang et al. (2019b)	2019	SUN	M	129S1/SvIm	18	*Y*	**N**	**N**
Yang et al. (2024)	2024	OSM	M	NR mice and SD rat	24 mice NR rat	*Y*	**N**	*Y*

Extracted data of rodent species, strains, and the number of animals used in studies investigating tyrosine kinase inhibitor (TKI)-induced cardiotoxicity and the type of experimental approach employed in each study. In vivo = TKI administered to live rodents, in vitro = primary cardiomyocytes, isolated from untreated rodents, ex vivo = intact cardiac tissues or organs were excised from untreated rodents and then exposed to a TKI under controlled laboratory conditions. Sprague–Dawley (SD), Wistar (W), Albino Wistar (W-Albino), Wistar Han RCC (HanRcc:W), Spontaneously Hypertensive Rat (SHR), Lewis (L), Dunkin Hartley (DH), Rat (R), Mouse (M), Guinea Pig (GP), not reported (NR), Yes (Y), No (N), Sorafenib (SOR), Sunitinib (SUN), Pazopanib (PZP), Gefitinib (GEF), Imatinib (IM), Bevacizumab (BEV), Crizotinib (CRI), Dasatinib (DAS), Lapatinib (LAP), Bosutinib (BOS), Nilotinib (NIL), Ponatinib (PON), Erlotinib (ERL), Osimertinib (OSM), Ibrutinib (IBR), Acalabrutinib (ACA), Vandetanib (VAN), Quizartinib (QZ), Lenvatinib (LEN), Alectinib (ALE), Regorafenib (REG) and Lestaurtinib (LST)

### Study characteristics

#### Type of rodent studies

From the 92 articles reporting outcomes of rodent testing TKI-induced cardiotoxicity, 63 studies used only in vivo models, 6 studies used only in vitro models, and 6 studies used only ex vivo models. Combination of in vivo and in vitro models was represented 14 times, the combination of in vivo and ex vivo was represented twice, and the combination of in vivo, in vitro and ex vivo models was only used once (Table 1). Overall, it was in vivo study models that were the predominant approach used to investigate TKI-induced cardiotoxicity.

#### Use of rodents in TKI-induced cardiotoxicity testing

Only three rodent species were identified as being used for TKI-induced cardiotoxicity testing. Fifty-one studies used rats, 46 studies used mice, and 4 studies used guinea pigs. Gerbils and hamsters were not used in any studies (Table 1).

Rats were the most frequently employed rodent model (51 studies) and were represented by multiple strains, including Sprague–Dawley, Wistar, Albino Wistar, Wistar Han RCC (HanRcc:W), Spontaneously Hypertensive Rat, and Lewis. Among these, the Sprague–Dawley strain was predominantly used (31/51 studies). The reported number of rats used within each study ranged from 12 (Bouitbir et al. 2019) to 114 (Aguirre et al. 2010). Twenty-one studies did not specify the number of rats used (Barr et al. 2014; Burke et al. 2019; Harvey and Leinwand 2015; Hasinoff et al. 2017; Jacob et al. 2016; Kobara et al. 2021; Li et al. 2023a; Liu et al. 2025, 2023; Ma et al. 2020b; Maharsy et al. 2014; McMullen et al. 2021; Monogiou Belik et al. 2024; Patyna et al. 2008; Pentassuglia et al. 2009; Sandhu et al. 2017; Shi et al. 2025; Stuhlmiller et al. 2017; Wolf et al. 2010, 2011). (Yang et al. 2024).

Mice represented the second most common rodent model used (46 studies), with the predominant strain being C57BL/6 (24/46 studies). Additional strains included C57BL/6NRJ, C57BL/6RJ, C57BL/J, FVB/N, 129S1/SvIm, ICR. Transgenic mouse colonies were used in five studies, including Gata4+/− (reduced cardiac stress resilience) and Bcl-2 overexpressing (apoptosis resistance) mice used by Maharsy et al. (2014), and BTKxid (defective BTK signalling), FYN−/− (loss of protective cardiac signalling), and αMHC-MerCreMerCskfl/fl mice (loss of Src regulation in cardiomyocytes) used by Xiao et al. (2020). In the 24/46 studies that reported the number of mice used, the total number ranged from 18 (Yang et al. 2019b) to 194 (Mozolevska et al. 2019).

Guinea pigs were only used in four studies, all of which used the Dunkin Hartley strain. Only one of these studies reported the number of guinea pigs used; 50 animals (Mooney et al. 2015).

Four studies out of the 92 failed to identify the strain of rodent used in experiments, all four of these studies only reported the use of mice (Chintalgattu et al. 2013; Duran et al. 2014; Yang et al. 2024, 2019a). Studies used a diverse age of rodents, ranging from neonates at 0–2 days old, to aged rodents up to 2 years old, but the majority of studies failed to report the age of animals used. The predominant number of studies (60/92) used only male rodents, with very few reporting the use of male and female rodents (8/92). Thirty-three studies failed to report the sex of the rodents used.

#### Tyrosine kinase inhibitors investigated in cardiotoxicity studies

Twenty-five TKIs were investigated in rodent models across the 92 studies, including, Sunitinib (SUN, 37 studies), Imatinib (IM, 18 studies), Sorafenib (SOR, 12 studies), Ibrutinib (IBR, 9 studies), getfitinib (GEF, 6 studies), ponatinib (PON, 6 studies), erlotinib (ERL, 4 studies), dasatinib (DAS, 3 studies), bosutinib (BOS, 2 studies), nilotinib (NIL, 2 studies), crizotinib (CRI, 2 studies), bevacizumab (BEV, 2 studies), pazopanib (PZP, 2 studies), vandetanib (VAN, 2 study), osimertinib (OSM, 2 study), lapatinib (LAP, 1 studies), lestaurtinib (LST, 1 study), quizartinib (QZ, 1 study), alectinib (ALE, 1 study), regorafenib (REG, 1 study), lenvatinib (LEN, 1 study), SIM01063 (1 study), PF-04254644 (1 study), PKI166 (1 study), and acalabrutinib (ACA, 1 study) (Table 1**)**. Overall, sunitinib was the most investigated TKI across the included rodent studies.

### Animal reports

Animal clinical outcomes were evaluated to determine the adverse effects of TKI treatment in rodent models, focusing on mortality, arrhythmias, and hypertension.

#### Mortality

Several papers reported elevated mortality rates associated with TKI treatment in rodent models (Aguirre et al. 2010; Alhazzani et al. 2024; Duran et al. 2014; Latifi et al. 2019; Li et al. 2023b; Patyna et al. 2008; Savi et al. 2018; Shuai et al. 2023). In studies involving sorafenib, mortality rates reached 55% at a dose of 30 mg/kg over two weeks (Duran et al. 2014) and were as high as 92.5% at 40 mg/kg over three weeks in mice (Li et al. 2022). In one study mortality rates in rats treated with sunitinib varied depending on the dose and duration of exposure, ranging from 2% at 0.3 mg/kg administered over six months to 30% at 15 mg/kg administered over 13 weeks (Patyna et al. 2008). In a study of imatinib, mortality rates in rats increased with dose, starting at 25% for the lowest dose of 50 mg/kg and reaching 100% for the highest dose of 200 mg/kg, with all treatments lasting three weeks (Savi et al. 2018). Similarly, a dose of 30 mg/kg of ponatinib administered to mice for one week resulted in a 20% mortality rate (Latifi et al. 2019). A 50% mortality rate was reported in rats after two weeks of dasatinib at a dose of 50 mg/kg (Alhazzani et al. 2024). The unmarketed TKI, PF-04254644 caused 40% mortality in rats when administered at a high dose of 320 mg/kg for one week (Aguirre et al. 2010). TKI treatment was associated with increased mortality rates in rodent models, particularly with sorafenib, sunitinib, imatinib, ponatinib, dasatinib, and PF-04254644, in a dose-dependent manner.

#### Arrythmias

Ibrutinib was identified as increasing the risk of atrial fibrillation (AF). The increased risk of AF was reported in mice treated with ibrutinib, 25 mg/kg over 4 weeks (Xiao et al. 2020) and 14 weeks (Jiang et al. 2019) and 10 mg/kg over 90 min (Tuomi et al. 2021). Whilst in rats, 25 mg/kg (Shuai et al. 2023) and 17 mg/kg (Shi et al. 2025; Yan et al. 2024) over 4 weeks of ibrutinib caused an increased risk of AF. After two weeks of 30 mg/kg of ibrutinib rats also had an increased risk to AF (Li et al. 2024b). In contrast, there was no increased risk of AF after alectinib treatment in rats treated with 10 mg/kg over 7–10 days (Liu et al. 2025).

QT prolongation was identified in pazopanib-treated rats (100 mg/kg) (Akman et al. 2014) as well as crizotinib-treated (40 mg/kg) (Cheng et al. 2023) and sunitinib-treated (40 mg/kg) mice (Li et al. 2023a). QT prolongation was also reported in mice treated with 30/kg of ibrutinib over 4 weeks. In an ex vivo Langendorff-perfused guinea pig heart, ≥ 10 µM of gefitinib induced a prolonged QT interval (Jie et al. 2021). QT prolongation was also identified after 2.4 and 24 µM of osimertinib in isolated guinea pigs hearts (Li et al. 2023b).

Ibrutinib treatment consistently increased the risk of atrial fibrillation in rats and mice, while QT prolongation was reported with pazopanib, crizotinib, ibrutinib sunitinib, gefitinib and osimertinib.

#### Hypertension

Hypertension or elevated blood pressure, was only reported with sunitinib, ibrutinib lenvatinib, crizotinib treatment in mice. Elevated blood pressure was reported in sunitinib-treated mice over a range of doses (10–40 mg/kg) (Blasi et al. 2012; Mohamad et al. 2024; Qin et al. 2024; Qu et al. 2025; Ren et al. 2021; Sourdon et al. 2021; Yang et al. 2019a, 2019b). Crizotinib (40 mg/kg) (Cheng et al. 2023), 4 mg/kg of lenvatinib (Krüger et al. 2025) and 30 mg/kg of ibrutinib (Li et al. 2024b) also raised blood pressure in mice.

### Physiological parameters

Physiological assays were employed to determine the direct effect of TKIs on rodent cardiovascular systems, including measurement of heart rate, stroke volume, cardiac output, fractional shortening, left ventricular mass and left ventricular internal dimension at diastole and systole.

#### Heart rate

Changes in heart rate were commonly reported within in vivo and ex vivo studies, with both increased and decreased heart rated observed depending on the TKI, the rodent model and for sunitinib, the age of the rat (Supplemental Table 9). PF-04254644 caused increased heart rate at 500 mg/kg for a single dose, 7-day study and at a 80 mg/kg over a 6-day study in rats (Aguirre et al. 2010). Crizotinib treatment at 40 mg/kg for 4 weeks significantly elevated heart rate in mice (Cheng et al. 2023), but reduced heart rate in guinea pigs (Jie et al. 2021).

Sorafenib treatment reduced heart rate in rats treated at 20 mg/kg for 4 weeks (Abdelgalil et al. 2020) and mice treated at 10 mg/kg for 3 weeks (French et al. 2010). Sorafenib-induced reduction in heart rate was also identified in two ex vivo rat studies (Henderson et al. 2013; Ma et al. 2020a). Alectinib was also identified as causing reduced HR in mice after 1 week of 10 mg/kg treatment (Liu et al. 2025).

Sunitinib treatment led to an increase in heart rate in rats treated at 25 mg/kg/day for 8 weeks (Blanca et al. 2016). Blasi et al. (2012) reported an increase in heart rate on day 31 of the on-treatment period, which then corrected at day 46 in the off-treatment period (Blasi et al. 2012). Conversely, Mooney et al. (2015) documented significant reduction in heart rate in guinea pigs treated with sunitinib at 16 mg/kg for 6 days. In mice there was an dose dependent effect on HR, with 10 mg/kg resulting in an increase in HR followed by an significant decline in HR at 20–40 mg/kg (Li et al. 2024a). Rats treated with sunitinib experienced an age-dependent decline with 3- and 12-month-old rats experiencing reduced heart rate after treatment, whilst at 24-months there was no significant change (Sandhu et al. 2017). An age-dependent decline in heart rate was also observed in sunitinib-treated rats, with 3-month-old and 12-month-old rats exhibiting significantly reduced heart rate, whereas this effect was absent in 24-month-old rats (Cooper et al. 2019).

Heart rate changes varied across TKIs, with both significant increases and decreases observed depending on the specific drug, the rodent model, and, in the case of sunitinib, the age of the rat.

#### Ejection fraction

TKI treatment was in the majority associated with reduced ejection fraction (EF) in rodent models (Supplemental Table 10). PF-04254644 treatment resulted in increased EF in rats following a single dose of 500 mg/kg and after 6 days of repeated dosing at 80 mg/kg (Aguirre et al. 2010). In contrast, EF was reduced in rats exposed to 80 mg/kg or 320/160 mg/kg for 7 days (Aguirre et al. 2010).

Sunitinib consistently reduced EF across multiple studies. Reduced EF was reported in mice treated with 40 mg/kg for 1 to 4 weeks (Bordun et al. 2015; Chintalgattu et al. 2013; Mozolevska et al. 2019; Qin et al. 2024; Ren et al. 2021; Stuhlmiller et al. 2017; Yang et al. 2019a). A reduced EF was also reported in rats treated with 25 mg/kg 3 times a week over 4 weeks (Mohamad et al. 2024). However, increased EF was observed in mice treated with 50 mg/kg for 5 weeks (Sourdon et al. 2021).

Sorafenib exposure also reduced EF in mice treated with 30 mg/kg for 2 weeks (Li et al. 2024c, 2022; Stuhlmiller et al. 2017), and in mice exposed to 30–40 mg/kg for 3 weeks with or without induced myocardial infarction (Duran et al. 2014).

Imatinib reduced EF in mice treated with 200 mg/kg for 5 weeks (Kerkelä et al. 2006) as did ponatinib in high-fat-diet-fed mice treated with 15 mg/kg for 2 weeks (Tousif et al. 2023), and in male and female mice treated with 30 mg/kg for 4 weeks, with the largest reduction in EF being in male mice (Madonna et al. 2021). Another drop was observed in mice treated with 30 mg/kg of ponatinib for 4 weeks (Mattii et al. 2024). Erlotinib treatment in rats at 10 mg/kg for 9 weeks also led to reduced EF (Mak et al. 2015). Reduced EF was observed in rats treated with ibrutinib (30 mg/kg) for 4 weeks (Li et al. 2024b). Bevacizumab exposure significantly reduced EF in mice treated with 10 mg/kg for 2 (Bordun et al. 2015) and 4 weeks (Mozolevska et al. 2019), and crizotinib significantly reduced EF in mice treated with 100 mg/kg for 6 weeks (Xu et al. 2024b). Lenvatinib at 4 mg/kg for 4 days also caused a reduction in EF in mice (Krüger et al. 2025). Regorafenib (200 mg/kg) for 6 weeks (Xu et al. 2024a) and osimertinib (25–50 mg/kg) for 3 weeks (Yang et al. 2024) also caused a significant reduction in EF.

Overall, the majority of TKIs were associated with reduced ejection fraction in rodent models. While one study reported a significant increase with sunitinib (Sourdon et al. 2021), most evidence indicated that sunitinib typically caused a decline (Bordun et al. 2015; Chintalgattu et al. 2013; Mohamad et al. 2024; Mozolevska et al. 2019; Qin et al. 2024; Ren et al. 2021; Stuhlmiller et al. 2017; Yang et al. 2019a).

#### Fractional shortening

Reduced fractional shortening (FS) was reported in multiple studies (Supplemental Table 11). Imatinib exposure consistently reduced FS in mice treated with 200 mg/kg for 5 weeks across several studies (Kerkelä et al. 2006; Kobara et al. 2021; Maharsy et al. 2014). Erlotinib treatment also reduced FS in rats treated with 10 mg/kg for 9 weeks (Mak et al. 2015). Crizotinib exposure for 6 weeks at 100 mg/kg also reduced FS in mice (Xu et al. 2024b).

Sorafenib was frequently associated with reduced FS in mice treated with 30 mg/kg for 2 weeks (Jensen et al. 2017a; Li et al. 2024c; Stuhlmiller et al. 2017) and in mice that had undergone either myocardial infarction (MI) induction or sham surgery and 30 mg/kg sorafenib treatment for 3 weeks (Duran et al. 2014). Reduced FS was also reported in rats following 4 weeks at 50 mg/kg (Liu et al. 2023).

Sunitinib caused notable reduction in FS in mice treated with 40 mg/kg for 2 to 4 weeks (Li et al. 2023a; Qin et al. 2024; Ren et al. 2021; Stuhlmiller et al. 2017; Yang et al. 2019a, 2019b). A similar reduction in FS was observed in rats treated with sunitinib (30 mg/kg) for 4 weeks (Mohamad et al. 2024). However, an increase in FS was reported in mice treated with 50 mg/kg for 6 weeks (Sourdon et al. 2021).

A reduction FS was observed in rats treated with ibrutinib (30 mg/kg) for 4 weeks (Li et al. 2024b). Regorafenib (200 mg/kg) for 6 weeks (Xu et al. 2024a) and osimertinib (25–50 mg/kg) for 3 weeks (Yang et al. 2024) also caused a significant reduction in FS in mice. Lenvatinib at 4 mg/kg for 4 days also caused reduced EF in mice (Krüger et al. 2025).

Ponatinib treatment also reduced FS in high-fat-diet-fed mice treated with 15 mg/kg for 2 weeks (Tousif et al. 2023) and in male and female mice treated with 30 mg/kg for 4 weeks (Madonna et al. 2021).

#### Stroke volume

Changes in stroke volume (SV) were observed in several studies (Supplemental Table 12). Ponatinib treatment reduced SV in mice treated with 30 mg/kg for 1 week (Latifi et al. 2019). Sorafenib exposure also reduced SV, with decrease observed in mice treated with 30 mg/kg for 2 weeks and in rats treated with 50 mg/kg for 4 weeks (Li et al. 2022) (Liu et al. 2023). Sunitinib exposure did not lead to significant changes in stroke volume in the studies identified (Blasi et al. 2012; French et al. 2010).

Conversely, increased SV was observed in rats treated with imatinib, with significant increase following treatment at 50 mg/kg for 8 weeks and 6 months (Heyen et al. 2013). Similarly, nilotinib treatment at both 40 mg/kg and 80 mg/kg for 4 weeks increased SV in rats (Wolf et al. 2011). PF-04254644 treatment led to increased SV in rats, with significant elevation reported after a single 500 mg/kg dose (measured on Day 6) and following 7 days of repeat dosing at 320/160 mg/kg (Aguirre et al. 2010).

Stroke volume changes varied across TKIs, with ponatinib and sorafenib associated with decreases, whereas imatinib, nilotinib, and PF-04254644 generally caused increased.

#### Cardiac output

Altered cardiac output (CO) was reported across several studies with differential effects (Supplemental Table 13). PF-04254644 treatment increased CO in rats following a single 500 mg/kg dose measured at days 6 and 12, as well as after 6 days of repeated dosing at 80 mg/kg (Aguirre et al. 2010). In contrast, sunitinib exposure at 40 mg/kg for 2 weeks reduced CO in mice (Li et al. 2023a). Similarly, erlotinib treatment in rats at 10 mg/kg for 9 weeks resulted in reduced CO (Mak et al. 2015). Osimertinib (25–50 mg/kg) for 3 weeks also caused a significant reduction in CO in mice (Yang et al. 2024).

#### Left ventricular mass

Left ventricular mass (LVM) was measured in rodent models, with observation of changes following treatment with sunitinib, sorafenib, and imatinib (Supplemental Table 14).

Significant changes in left ventricular mass (LVM) were observed following sunitinib, sorafenib, and imatinib treatments in rodent models. Exposure to sunitinib increased LVM in mice treated with 50 mg/kg for 6 weeks (Sourdon et al. 2021), while reduced LVM was reported in female mice treated with 40 mg/kg for 4 weeks (Harvey and Leinwand 2015).

Sorafenib treatment increased LVM in mice treated with 30 mg/kg for 2 weeks (Jensen et al. 2017a). Imatinib also reduced LVM in young mice treated with 200 mg/kg for 5 weeks (Maharsy et al. 2014), but caused an increase in rats treated with 50 mg/kg for 8 weeks (Heyen et al. 2013).

Sunitinib and erlotinib treatment reduced cardiac output in rodent models, whereas PF-04254644 caused an increase dependent on dose and duration.

#### Left ventricular wall thickness

Changes in left ventricular wall thickness following TKI treatment were variable, with both increased and reduced thickness measured depending on the TKI and study design (Supplemental Table 15).

Sunitinib treatment at 40 mg/kg for 4 weeks resulted in reduced left ventricular thickness in female mice (Harvey and Leinwand 2015). However, increased thickness was also reported in mice after 4 weeks at the same dose (Harvey and Leinwand 2015; Ren et al. 2021).

Imatinib exposure reduced left ventricular thickness in both old and young mice treated at 200 mg/kg for 5 weeks (Maharsy et al. 2014). However, increased thickness was observed in rats treated with 50–100 mg/kg for 3 weeks (Savi et al. 2018), and 50 mg/kg for 8 weeks or 6 months (Heyen et al. 2013).

Nilotinib treatment increased left ventricular thickness after 4 weeks at both 40 mg/kg and 80 mg/kg in rats (Wolf et al. 2011), whilst Erlotinib exposure caused it to be reduced in rats treated with 10 mg/kg for 9 weeks (Mak et al. 2015).

Changes in left ventricular wall thickness following TKI treatment were variable, with both increases and decreases observed depending on the specific TKI and study design.

#### Left ventricular internal dimension at diastole and systole

Changes in left ventricular internal dimension at diastole (LVIDd) and systole (LVIDs) were observed in several studies investigating TKI-induced cardiotoxicity in rodent models (Supplemental Table 16). Sunitinib treatment resulted in increased LVIDd in mice following treatment for 2 weeks (Stuhlmiller et al. 2017) and 4 weeks (Mozolevska et al. 2019) at 40 mg/kg and increased LVIDs after 2 (Jensen et al. 2017b) and 4 weeks (Ren et al. 2021) at the same dose. Imatinib exposure produced mixed effects. LVIDd increased in older mice after 5 weeks at 200 mg/kg (Maharsy et al. 2014), while LVIDd and LVIDs both decreased following 5 weeks at the same dose in a Kerkelä et al.’s study (Kerkelä et al. 2006). Bevacizumab treatment for 4 weeks at 10 mg/kg significantly increased LVIDd in mice (Mozolevska et al. 2019). With lenvatinib treatment in mice there was a significant increase in both LVIDd and LVIDd (Krüger et al. 2025). Ponatinib treatment increased LVIDd in high-fat-diet-fed mice after 2 weeks at 15 mg/kg, though no changes were reported in mice on a normal diet (Tousif et al. 2023). Ponatinib also induced a reduction in both LVIDd and LVIDs after 30 mg/kg for 4 weeks (Mattii et al. 2024). Erlotinib was linked to increased LVIDs in mice after 2 weeks at 50 mg/kg (Jensen et al. 2017b), while LVIDd and LVIDs both decreased in rats treated with 10 mg/kg for 9 weeks (Mak et al. 2015). Sorafenib treatment reduced LVIDs in rats after 4 weeks at 50 mg/kg (Liu et al. 2023).

Sunitinib, bevacizumab lenvatinib, and ponatinib were consistently associated with increases in left ventricular internal dimensions, while sorafenib treatment led to reductions. Mixed effects were observed for imatinib and erlotinib, depending on rodent model, age (imatinib-only) and dosing conditions.

### Histopathological findings

Histopathological assessment identified fibrotic changes, inflammation, necrosis and other common features associated with cardiotoxicity following dosing with TKIs.

#### Gross structural features

Disruption of cellular and tissue organisation was frequently reported in studies of sorafenib**,** imatinib, ibrutinib, sunitinib, crizotinib and bevacizumab in in vivo rats and mice. Observations included sarcomere disorganisation, misaligned of myofilaments, loss of myofibril integrity, Z-band disruption, and disorganized myocardium (Abdelgalil et al. 2020; Aguirre et al. 2010; Li et al. 2022; Liu et al. 2023; Ma et al. 2020a; Maayah et al. 2014; Marslin et al. 2015; Mozolevska et al. 2019; Savi et al. 2018; Shuai et al. 2023; Song et al. 2022; Xu et al. 2022, 2024b).

Myofibrillar loss also occurred in in vitro rat myocytes treated with 2 µM bosutinib, 2 µM ponatinib, and 30 µM imatinib over 72 h (Hasinoff et al. 2017; Herman et al. 2011). Loss of myofibrils was associated with imatinib-treated myocytes. Structural disorganisation was also reported in isolated rat cardiac fibroblasts with sunitinib (3 µM) and imatinib (10 µM), causing a loss of spindle-like morphology and reduced membrane protrusions (Burke et al. 2019). Sarcomeric disorganisation, Z-line condensation and misalignment was identified following treatment of a tissue engineered 3D in vitro model of primary rat cardiomyocytes with gefitinib (1 μM), imatinib (10 μM), lapatinib (15 μM), sunitinib (1 μM), lestaurtinib (10 μM), sorafenib (10 μM), and vandetanib (10 μM), as well as for erlotinib (100 μM) and dasatinib (10 μM) (Jacob et al. 2016). Structural changes such as myofibrillar damage was also induced by PKI166 in adult rat cardiomyocytes (Pentassuglia et al. 2009).

Structural changes to mitochondria also emerged as a central effect of TKI treatment, with evidence of biogenesis (increased numbers of mitochondria), swollen and enlarged mitochondria, disruption to or loss of cristae was identified in sorafenib, sunitinib, imatinib, ibrutinib, osimertinib and gefitinib-treated rats and mice (imatinib only) (French et al. 2010; Jacob et al. 2016; Kerkelä et al. 2006; Li et al. 2024b; Liu et al. 2023; Ma et al. 2020a; Maharsy et al. 2014; Savi et al. 2018; Yang et al. 2024).

Cardiac hypertrophy and enlarged myocytes were frequently observed in sorafenib-treated mice (Duran et al. 2014) (Li et al. 2022), sunitinib-treated rats (Mohamad et al. 2024), sorafenib-treated rats (Liu et al. 2023), gefitinib-treated rats (Korashy et al. 2016), regorafenib-treated mice (Xu et al. 2024a), imatinib-treated mice (Kobara et al. 2021), and ibrutinib-treated mice (Shuai et al. 2023) (Yan et al. 2024). Cardiac hypertrophy was also identified in isolated neonatal rat ventricular myocytes following 12-h exposure to 2 µM imatinib. This increase in cardiac volume was not observed at the increased concentration of 5 µM, although cardiomyocytes appeared visually larger (Barr et al. 2014). Hypertrophy was also identified in rat cardiac fibroblasts treated with > 1 µM of sunitinib (McMullen et al. 2021).

Evidence of histopathological markers of cellular stress were present in rats and mice treated with sorafenib (Li et al. 2022), sunitinib (Li et al. 2024a; Maharsy et al. 2014), gefitinib (AlAsmari et al. 2020; Korashy et al. 2016) and imatinib (Abdel-Wahab et al. 2025; Herman et al. 2014, 2011; Wolf et al. 2010). Hyperchromasia was induced in rats treated with 30 mg/kg of gefitinib over 21 days (AlAsmari et al. 2020) whilst the same dose of gefitinib over the same number of days caused scattered nuclei as reported in rats (Korashy et al. 2016). Sorafenib (30 mg/kg) induced nuclear fragmentation in mice cardiomyocytes after 2 weeks of treatment (Li et al. 2022). Imatinib was found to cause cytoplasmic vacuolisation in rat myocytes at 10–50 mg/kg over 10 days, at 50 and 100 mg/kg over 14 days (Herman et al. 2011), at 50–200 mg/kg over 4 weeks (Herman et al. 2014) and at 40 mg/kg over 4 weeks (Abdel-Wahab et al. 2025). Imatinib treatment in mice over 4 weeks at 180 mg/kg induced cytoplasmic inclusion bodies and the generation of lysosomes in rat heart tissue (Wolf et al. 2010). Cytoplasmic vacuolisation was also induced within mice treated with sunitinib, 100 mg/kg for 1 month (Maharsy et al. 2014) and 10–40 mg/kg over 2 weeks (Li et al. 2024a).

TKI treatment frequently induced histopathological changes, with structural abnormalities in rodents such as myofibrillar disorganisation, mitochondrial damage, and hypertrophy, reflecting cellular stress.

#### Fibrosis

Fibrotic changes were reported across multiple TKIs, including sorafenib, sunitinib, ibrutinib, imatinib, dasatinib, ponatinib and PF-04254644 in both rat and mice models. Fibrosis was present in sunitinib-treated rats with 25 mg/kg over 4 weeks (Mohamad et al. 2024; Sayed-Ahmed et al. 2019) and 8 weeks (Blanca et al. 2016), 40 mg/kg over 4 weeks (Qu et al. 2025). Whilst mice experienced cardiac fibrosis at 40 mg/kg over 32 days (Xu et al. 2022) and 4 weeks (Qin et al. 2024) and at 50 mg/kg over 6 weeks (Sourdon et al. 2021).

Sorafenib induced fibrosis in both mice and rats, at 30 mg/kg for 2 weeks (Li et al. 2024c, 2022) and 50 mg/kg at 4 weeks (Ma et al. 2020a). TKI-induced fibrosis was also reported in ponatinib-treated mice (Madonna et al. 2022; Tousif et al. 2023). Imatinib treatment (25–100 mg/kg) in mice (Song et al. 2022) and rats (Abdel-Wahab et al. 2025), and nilotinib (40–80 mg/kg) (Wolf et al. 2011) and PF-04254644 (120–320 mg/kg) (Aguirre et al. 2010) exposure in rats also induced cardiac fibrosis. Cardiac fibrosis was also reported in mice treated with ibrutinib, at 25–30 mg/kg over 4 weeks (Li et al. 2025; Shuai et al. 2023; Xiao et al. 2020) and over 14 weeks (Jiang et al. 2019). Rats also experienced cardiac fibrosis after ibrutinib treatment (Li et al. 2024b; Yan et al. 2024) and after 2 weeks of 50 mg/kg of dasatinib (Alhazzani et al. 2024).

#### Inflammation and immune cell infiltration of tissues

Inflammation, often accompanied by immune cell infiltration of tissues, was frequently reported in response to TKI treatment. Imatinib was reported to induce inflammation and immune cell infiltration in both rats (Abdel-Wahab et al. 2025; Herman et al. 2011; Wolf et al. 2010) and mice (Song et al. 2022). Immune cell infiltration and inflammation in response to sunitinib was consistently reported in rats (Mohamad et al. 2024; Sayed-Ahmed et al. 2019) and mice (Qin et al. 2024) following 4-weeks of treatment. Sorafenib (Aguirre et al. 2010; Li et al. 2024c) and Ponatinib (Tousif et al. 2023) treatment in mice, along with PF-04254644 (Abdelgalil et al. 2020) exposure in rats, also triggered inflammatory responses in cardiac tissue. Additionally, vandetanib treatment in mice (Cosgun et al. 2021), nilotinib treatment in rats (Wolf et al. 2011) and dasatinib treatment in rats (Alhazzani et al. 2024) were all linked to also cardiac inflammation.

#### Necrosis

Necrotic myocardial damage was also reported as a histopathological biomarker of TKIs. Imatinib consistently induced necrosis in both rats and mice. Herman et al. (2011, 2014) reported myocardial necrosis at doses of 50–200 mg/kg over 10 days and 2 weeks (Herman et al. 2011) and 4 weeks in rats (Herman et al. 2014). Similarly, Maharsy et al. (2014) found age-dependent necrosis in mice treated with 200 mg/kg for 5 weeks, with older animals exhibiting more pronounced damage (Maharsy et al. 2014). Wolf et al. (2010) further identified necrotic damage in the hearts of rats treated with Imatinib at 120–180 mg/kg for 4 weeks (Wolf et al. 2010). Sorafenib caused myocardial necrosis in multiple studies. Li et al. (2022) observed cardiomyocyte necrosis in mice treated with 30 mg/kg for 2 weeks, while Ma et al. (2020a, b) documented significant necrotic damage in rats treated with 50 mg/kg for 4 weeks (Li et al. 2022; Ma et al. 2020a). Dasatinib at 50 mg/kg over weeks also induced myocardial necrosis in rats (Alhazzani et al. 2024), vandetanib at 25 mg/kg treatment of rats over a period of one month was associated with severe myocardial necrosis (Cosgun et al. 2021). Gefitinib also induced necrotic changes, with Korashy et al. (2016) reporting myocardial degeneration in rats treated with 20–30 mg/kg for 3 weeks (Korashy et al. 2016). Necrotic cardiac fibroblasts were also present following treatment with 10 µM of sunitinib (McMullen et al. 2021).

### Biomarker analysis

The measurement of cardiac biomarkers is often used in pre-clinical and clinical trials to detect drug-induced cardiotoxicity. Within the identified dataset of studies significant alterations in levels of cardiac troponin, creatine kinase and brain natriuretic peptide-32 (BNP) were reported in response to TKI treatment within rodents (Supplemental Tables 17–19).

#### Troponin (cTnI and cTnT)

Cardiac troponin I (cTnI) and cardiac troponin T (cTnT) were commonly used as measures of myocardial injury, with several studies identifying increased expression in response to TKIs (Supplemental Table 17).

Increased cTnI was observed following gefitinib treatment in rats after exposure to 30 mg/kg/day for 2 weeks (Alanazi et al. 2022) and 3 weeks (AlAsmari et al. 2020; Alhoshani et al. 2020). Sunitinib also resulted in increased troponin in rats treated with 25 mg/kg for 5 weeks (Aldemir et al. 2020) and in mice treated with 7.5 mg/kg for 2 weeks (Bouitbir et al. 2019). Increased troponin was observed in mice following 4 weeks of treatment at 40 mg/kg (Qin et al. 2024; Ren et al. 2021) and after 32 days at the same dose (Xu et al. 2022). Troponin levels were also increased in mice treated with 40 mg/kg for 2 weeks (Bordun et al. 2015). Imatinib treatment resulted in increased cTnI in rats treated with 200 mg/kg for 4 weeks, while lower doses (50–100 mg/kg) showed no significant change (Herman et al. 2014). However, 40 mg/kg over 4 weeks in another study did cause a significant decline in cTnI in rats (Abdel-Wahab et al. 2025). Osimertinib (25–50 mg/kg) over 3 weeks also caused a significant increase in cTnI in mice (Yang et al. 2024). Ponatinib treatment led to increased troponin levels in both male and female mice treated at 30 mg/kg for 4 weeks, with a greater increase observed in males (Madonna et al. 2021). Increased troponin levels were also reported in rats treated with vandetanib at 25 mg/kg for 1 month (Cosgun et al. 2021) and in mice treated with bevacizumab at 10 mg/kg for 15 days (Bordun et al. 2015).

#### Creatine kinase (CK and CK-MB)

Creatine kinase (CK) and its isoform CK-MB were other biomarkers of cardiotoxicity, both of which were frequently increased in response to TKI treatment, though some studies reported a decreased in response to dasatinib, gefitinib, lapatinib, imatinib, and sorafenib (Supplemental Table 18).

Increased CK-MB was reported following gefitinib treatment in rats after 3 weeks of exposure to 30 mg/kg/day (Alanazi et al. 2022; AlAsmari et al. 2020). Significant increase in CK-MB were also seen with sunitinib, including in rats treated with 25 mg/kg for 3 (Imam et al. 2020) to 4 weeks (Mohamad et al. 2024; Sayed-Ahmed et al. 2019) and in mice treated with 40 mg/kg for 4 weeks (Qin et al. 2024). A significant increase in both CK and CK-MB was observed in rats given 100 mg/kg sunitinib for 1 month (Maayah et al. 2014).

Increased CK-MB was also observed in vandetanib-treated rats (25 mg/kg for 1 month) (Cosgun et al. 2021) and in crizotinib-treated mice following 6 weeks of exposure to 100 mg/kg (Xu et al. 2024b). Mice treated with sorafenib at 30 mg/kg for 2 weeks also showed increased CK-MB (Li et al. 2024c, 2022). Osimertinib also induced an increase in CK-MB in mice treated with 25–50 mg/kg over 3 weeks (Yang et al. 2024).

Increase in total CK was noted in sunitinib-treated mice after 2 weeks at 7.5 mg/kg (Bouitbir et al. 2019) and in rats after 6 months at 6 mg/kg (Patyna et al. 2008). Increased CK was also observed in mice treated with imatinib at 50 and 100 mg/kg for 2 weeks in each of Song et al.’s studies (Song et al. 2022, 2023). This increase was also reflected in CK-MB after imatinib treated at 40 mg/kg for 4 weeks in rats (Abdel-Wahab et al. 2025).

Increased CK was reported with erlotinib, gefitinib, lapatinib, sunitinib, imatinib, sorafenib, vandetanib, and lestaurtinib at concentrations ranging from 1 to 150 µM when applied to a tissue-engineered 3D in vitro model of primary rat cardiomyocytes embedded in a fibrin hydrogel (Jacob et al. 2016). In contrast, dasatinib, gefitinib, lapatinib, imatinib, and sorafenib reduced CK at lower concentrations (Jacob et al. 2016). Interestingly, a decrease in total CK was identified by Liu et al (2023) in sorafenib treated rats after a 14 week exposure (Liu et al. 2023).

Creatine kinase and CK-MB levels were frequently elevated following TKI treatment, although a few studies reported reductions in CK with dasatinib, gefitinib, lapatinib, imatinib, and sorafenib treatment.

#### Brain natriuretic peptide-32 (BNP)

Brain natriuretic peptide-32 (BNP) and its inactive precursor NT-proBNP are established biomarkers of cardiac stress and injury. Multiple studies identified a significant increase in response to TKIs (Supplemental Table 19).

Increased BNP was observed following gefitinib treatment in rats after exposure to 30 mg/kg for 3 weeks (Korashy et al. 2016). Similarly, sunitinib exposure resulted in increased BNP levels in rats treated with 25 mg/kg for 3 weeks (Imam et al. 2020) and in those exposed to 100 mg/kg for 1 month (Maayah et al. 2014). BNP levels were also elevated in neonatal rat ventricular myocytes (NRVMs) and in female adult rat ventricular myocytes (ARVMs) treated with 150 ng/ml sunitinib for 36 h (Harvey and Leinwand 2015). Sorafenib exposure in mice (30 mg/kg for 2 weeks) resulted in elevated BNP levels (Li et al. 2022).

Increased NT-proBNP, the inactive precursor of BNP, was reported following gefitinib treatment in rats (30 mg/kg for 3 weeks) (Alanazi et al. 2022; AlAsmari et al. 2020). Increased NT-proBNP levels were also reported in mice treated with sunitinib at 40 mg/kg for 4 weeks (Ren et al. 2021).

### Risk of bias assessment

The quality of in vivo and ex vivo TKI rodent toxicity studies was evaluated using an adapted SYRCLE's tool. The risk of bias was categorised as"Low,"indicating a low risk of bias;"Unclear,"reflecting insufficient information to determine the risk of bias; and"High,"denoting a high risk of bias. Assessments were conducted across several domains, including sequence generated, baseline characteristics, allocation concealment, random housing, treatment blinding, random outcome selection, result blinding, incomplete outcome data, selective outcome reporting, and other sources of bias. Details of the assessments are summarised in Table 2 for in vivo studies and in Table 3 for ex vivo studies.

**Table 2:** Risk bias for in vivo tyrosine kinase inhibitor (TKI) rodent toxicity studies using an adapted SYRCLE’s Tool

References	Sequence generated	Baseline characteristics	Allocation concealment	Random housing	Treatment blinding	Random outcome selection	Result blinding	Incomplete outcome data	Selective outcome reporting	Other sources of bias
Abdelgalil et al. (2020)	Unclear	Low	Unclear	Unclear	Unclear	Unclear	Unclear	Unclear	Low	Low
Abdel-Wahab et al. (2025)	Low	Low	Unclear	Unclear	Unclear	Unclear	Unclear	Unclear	Low	Low
Aguirre et al. (2010)	Unclear	Low	Unclear	Unclear	Unclear	Unclear	Unclear	Unclear	Low	Low
Akman et al. (2014)	Low	Low	Unclear	Unclear	Unclear	Unclear	Unclear	Unclear	Low	Low
Alanazi et al. (2022)	Unclear	Low	Unclear	Unclear	Unclear	Unclear	Unclear	Unclear	Low	Low
AlAsmari et al. (2020)	Low	Low	Unclear	Unclear	Unclear	Unclear	Unclear	Unclear	Low	Low
Aldemir et al. (2020)	Unclear	Low	Unclear	Unclear	Unclear	Unclear	Low	Unclear	Low	Low
Alhazzani et al. (2024)	Low	Low	Unclear	Unclear	Unclear	Unclear	Unclear	Unclear	Low	Low
Alhoshani et al. (2020)	Low	Low	Unclear	Unclear	Unclear	Unclear	Unclear	Unclear	Low	Low
Blanca et al. (2016)	Unclear	Low	Unclear	Unclear	Unclear	Unclear	Unclear	Unclear	Low	Low
Blasi et al. (2012)	Unclear	Low	Unclear	Unclear	Unclear	Unclear	Unclear	Unclear	Low	Low
Bordun et al. (2015)	Low	Low	Unclear	Unclear	Unclear	Unclear	Low	Unclear	Low	Low
Bouitbir et al. (2019)	Low	Low	Unclear	Unclear	Unclear	Unclear	Unclear	Unclear	Low	Low
Cheng et al. 2023	Low	Low	Unclear	Unclear	Unclear	Unclear	Unclear	Unclear	Low	Low
Chintalgattu et al. (2013)	Unclear	Low	Unclear	Unclear	Unclear	Unclear	Low	Unclear	Low	Low
Cosgun et al. (2021)	Unclear	Low	Unclear	Unclear	Unclear	Unclear	Low	Unclear	Low	Low
Duran et al. (2014)	Unclear	Low	Unclear	Unclear	Unclear	Low	Unclear	Unclear	Low	Low
French et al. (2010)	Unclear	Low	Unclear	Unclear	Unclear	Unclear	Unclear	Unclear	Low	Low
Harvey and Leinwand (2015)	Unclear	Low	Unclear	Unclear	Unclear	Unclear	Unclear	Unclear	Low	Low
Herman et al. (2011)	Unclear	Low	Unclear	Unclear	Unclear	Unclear	Low	Unclear	Low	Low
Herman et al. (2014)	Unclear	Low	Unclear	Unclear	Unclear	Unclear	Low	Unclear	Low	Low
Heyen et al. 2013	Low	Low	Unclear	Unclear	Unclear	Unclear	Unclear	Unclear	Low	Low
Imam et al. (2020)	Low	Low	Unclear	Unclear	Unclear	Unclear	Unclear	Unclear	Low	Low
Jensen et al. (2017a)	Unclear	Low	Unclear	Unclear	Unclear	Unclear	Unclear	Unclear	Low	Low
Jensen et al. (2017b)	Unclear	Low	Unclear	Unclear	Unclear	Unclear	Unclear	Unclear	Low	Low
Jiang et al. (2019)	Unclear	Low	Unclear	Unclear	Unclear	Unclear	Unclear	Unclear	Low	Low
Kerkelä et al. (2006)	Unclear	Low	Unclear	Unclear	Unclear	Unclear	Unclear	Unclear	Low	Low
Kobara et al. (2021)	Unclear	Low	Unclear	Unclear	Unclear	Unclear	Unclear	Unclear	Low	Low
Korashy et al. (2016)	Low	Low	Unclear	Unclear	Unclear	Unclear	Unclear	Unclear	Low	Low
Krüger et al. (2025)	Low	Low	Unclear	Unclear	Unclear	Unclear	Unclear	Unclear	Low	Low
Latifi et al. (2019)	Unclear	Low	Unclear	Unclear	Unclear	Unclear	Unclear	Unclear	Low	Low
Li et al. (2019)	Unclear	Low	Unclear	Unclear	Unclear	Unclear	Unclear	Unclear	Low	Low
Li et al. (2022)	Low	Low	Unclear	Unclear	Unclear	Unclear	Low	Unclear	Low	Low
Li et al. (2023a)	Unclear	Low	Unclear	Unclear	Unclear	Unclear	Unclear	Unclear	Low	Low
Li et al. (2024a)	Low	Low	Unclear	Unclear	Unclear	Unclear	Unclear	Unclear	Low	Low
Li et al. (2024b)	Unclear	Low	Unclear	Unclear	Unclear	Unclear	Low	Unclear	Low	Low
Li et al. (2024c)	Unclear	Low	Unclear	Unclear	Unclear	Unclear	Unclear	Unclear	Low	Low
Li et al. (2025)	Unclear	Low	Unclear	Unclear	Unclear	Unclear	Low	Unclear	Low	Low
Lim et al. (2010)	Low	Low	Unclear	Unclear	Unclear	Unclear	Low	Unclear	Low	Low
Liu et al. (2023)	Unclear	Low	Unclear	Unclear	Unclear	Unclear	Low	Unclear	Low	Low
Liu et al. (2024)	Unclear	Low	Unclear	Unclear	Unclear	Unclear	Unclear	Unclear	Low	Low
Liu et al. (2025)	Unclear	Low	Unclear	Unclear	Low	Unclear	Low	Unclear	Low	Low
Ma et al. (2020a)	Low	Low	Unclear	Unclear	Unclear	Unclear	Unclear	Unclear	Low	Low
Maayah et al. (2014)	Low	Low	Unclear	Unclear	Unclear	Unclear	Unclear	Unclear	Low	Low
Madonna et al. (2021)	Low	Low	Unclear	Unclear	Unclear	Unclear	Low	Unclear	Low	Low
Madonna et al. (2022)	Low	Low	Unclear	Unclear	Unclear	Unclear	Low	Unclear	Low	Low
Maharsy et al. (2014)	Unclear	Low	Unclear	Unclear	Unclear	Unclear	Unclear	Unclear	Low	Low
Mak et al. (2015)	Unclear	Low	Unclear	Unclear	Unclear	Unclear	Unclear	Unclear	Low	Low
Mao et al. (2012)	Low	Low	Unclear	Unclear	Unclear	Unclear	Unclear	Unclear	Low	Low
Marslin et al. (2015)	Unclear	Low	Unclear	Unclear	Unclear	Unclear	Unclear	Unclear	Low	Low
Mattii et al. (2024)	Low	Low	Unclear	Unclear	Unclear	Unclear	Low	Unclear	Low	Low
Mohamad et al. (2024)	Low	Low	Unclear	Unclear	Unclear	Unclear	Low	Unclear	Low	Low
Monogiou Belik et al. (2024)	Low	Low	Unclear	Unclear	Unclear	Unclear	Low	Unclear	Low	Low
Mooney et al. (2015)	Unclear	Low	Unclear	Unclear	Unclear	Unclear	Unclear	Unclear	Low	Low
Mozolevska et al. (2019)	Low	Low	Unclear	Unclear	Unclear	Unclear	Low	Unclear	Low	Low
Patyna et al. (2008)	Unclear	Low	Unclear	Unclear	Unclear	Unclear	Unclear	Unclear	Low	Low
Qin et al. (2024)	Low	Low	Unclear	Unclear	Unclear	Unclear	Unclear	Unclear	Low	Low
Qu et al. (2025)	Unclear	Low	Unclear	Unclear	Unclear	Unclear	Unclear	Unclear	Low	Low
Ren et al. (2021)	Low	Low	Unclear	Unclear	Unclear	Unclear	Unclear	Unclear	Low	Low
Savi et al. (2018)	Unclear	Low	Unclear	Unclear	Unclear	Unclear	Unclear	Unclear	Low	Low
Sayed-Ahmed et al. (2019)	Low	Low	Unclear	Unclear	Unclear	Unclear	Unclear	Unclear	Low	Low
Shi et al. (2025)	Unclear	Low	Unclear	Unclear	Unclear	Unclear	Unclear	Unclear	Low	Low
Shuai et al. (2023)	Low	Low	Unclear	Unclear	Unclear	Unclear	Low	Unclear	Low	Low
Song et al. (2022)	Unclear	Low	Unclear	Unclear	Unclear	Unclear	Unclear	Unclear	Low	Low
Song et al. (2023)	Low	Low	Unclear	Unclear	Unclear	Unclear	Unclear	Unclear	Low	Low
Sourdon et al. (2021)	Low	Low	Unclear	Unclear	Unclear	Unclear	Unclear	Unclear	Low	Low
Stuhlmiller et al. (2017)	Unclear	Low	Unclear	Unclear	Unclear	Unclear	Low	Unclear	Low	Low
Tousif et al. (2023)	Low	Low	Unclear	Unclear	Unclear	Unclear	Unclear	Unclear	Low	Low
Tuomi et al. (2018)	Low	Low	Unclear	Unclear	Unclear	Unclear	Unclear	Unclear	Low	Low
Tuomi et al. (2021)	Unclear	Low	Unclear	Unclear	Unclear	Unclear	Unclear	Unclear	Low	Low
Wolf et al. (2010)	Unclear	Low	Unclear	Unclear	Unclear	Unclear	Unclear	Unclear	Low	Low
Wolf et al. (2011)	Low	Low	Unclear	Unclear	Unclear	Unclear	Unclear	Unclear	Low	Low
Xiao et al. (2020)	Unclear	Low	Unclear	Unclear	Unclear	Unclear	Unclear	Unclear	Low	Low
Xu et al. (2022)	Low	Low	Unclear	Unclear	Unclear	Unclear	Unclear	Unclear	Low	Low
Xu et al. (2024a)	Unclear	Low	Unclear	Unclear	Unclear	Unclear	Unclear	Unclear	Low	Low
Xu et al. (2024b)	Unclear	Low	Unclear	Unclear	Unclear	Unclear	Unclear	Unclear	Low	Low
Yan et al. (2024)	Low	Low	Unclear	Unclear	Unclear	Unclear	Unclear	Unclear	Low	Low
Yang et al. (2019a)	Unclear	Low	Unclear	Unclear	Unclear	Unclear	Unclear	Unclear	Low	Low
Yang et al. (2019b)	Low	Low	Unclear	Unclear	Unclear	Unclear	Unclear	Unclear	Low	Low
Yang et al. (2024)	Low	Low	Unclear	Unclear	Unclear	Unclear	Unclear	Unclear	Low	Low

Risk was categorised as “Low”, “Unclear” or “High” for reliability and potential bias of each study under each domain, sequence generated, baseline characteristics, allocation concealment, random housing, treatment blinding, random outcome selection, result blinding, incomplete outcome data, selective outcome reporting and other sources of bias

**Table 3:** Risk Bias of Ex Vivo Tyrosine Kinase Inhibitor (TKI) Rodent Toxicity Studies Using an Adapted SYRCLE’s Tool

References	Sequence generation	Baseline characteristics	Allocation concealment	Random housing	Treatment blinding	Random outcome selection	Result blinding	Incomplete outcome data	Selective outcome reporting	Other sources of bias
Blasi et al. (2012)	Unclear	Low	Unclear	Unclear	Unclear	Unclear	Unclear	Unclear	Low	Low
Cooper et al. (2019)	Low	Low	Unclear	Unclear	Unclear	Unclear	Low	Unclear	Low	Low
Henderson et al. (2013)	Unclear	Low	Unclear	Unclear	Unclear	Unclear	Unclear	Unclear	Low	Low
Jie et al. (2021)	Unclear	Low	Unclear	Unclear	Unclear	Unclear	Unclear	Unclear	Low	Low
Kuburas et al. (2022)	Low	Low	Unclear	Unclear	Unclear	Unclear	Unclear	Unclear	Low	Low
Li et al. (2023b)	Low	Low	Unclear	Unclear	Unclear	Unclear	Unclear	Unclear	Low	Low
Liu et al. (2025)	Unclear	Low	Unclear	Unclear	Low	Unclear	Low	Unclear	Low	Low
Ma et al. (2020a)	Unclear	Low	Unclear	Unclear	Unclear	Unclear	Unclear	Unclear	Low	Low
Sandhu et al. (2017)	Low	Low	Unclear	Unclear	Unclear	Unclear	Low	Unclear	Low	Low

The quality of the ex vivo TKI rodent toxicity studies was evaluated using an adapted SYRCLE’s tool. Risk was categorised as “Low”, “Unclear” or “High” to reflect the reliability and potential bias of each study under each domain, sequence generation, baseline characteristics, allocation concealment, random housing, treatment blinding, random outcome selection, result blinding, incomplete outcome data, selective outcome reporting and other sources of bias

#### Risk of bias analysis for in vivo studies

Eighty studies were identified as in vivo experiments (Table 2). For sequence generation, 37 studies were classified as having a low risk of bias, while 43 studies were identified as unclear due to insufficient reporting on randomisation methods. Baseline characteristics showed strong performance, with all 80 studies identified as having a low risk of bias, indicating that groups were adequately comparable at baseline. In contrast, no studies demonstrated a low risk of bias for allocation concealment, and random housing, as these domains were consistently identified as unclear across all 80 studies, indicating a lack of transparency in describing methods to minimise selection and performance biases. Only one paper identified as low risk for treatment blinding. For random outcome selection, 1 study was classified as low risk with the remaining 79 identified as unclear. For result blinding, only 20 studies were classified as low risk, whereas the remaining 60 studies were identified as unclear, highlighting the frequent absence of adequate blinding measures during outcome assessments. For incomplete outcome data, no studies were identified as low risk. Selective outcome reporting was identified as low risk in all 80 studies, suggesting consistent reporting of intended outcomes. Similarly, other sources of bias were uniformly identified as low risk across all studies, indicating no significant concerns in these areas.

None of the in vivo studies were identified as having a low risk of bias across all assessed domains. The highest number of domains identified as “Low” in a single study was five, which included sequence generation, baseline characteristics, result blinding, selective outcome reporting, and other sources of bias. Eleven studies were rated as"Low"in these domains: Bordun et al. (2015), Li et al. (2022), Lim et al. (2010), Liu et al. (2025), Madonna et al. (2021 and 2022), Mattii et al. (2024), Mohamad et al. (2024), Monogiou Belik et al. (2024), Mozolevska et al. (2019), and Shuai et al. (2023).

#### Risk of bias analysis for ex vivo studies

Nine studies were included in analysis of ex vivo studies (Table 3). For sequence generated, 4 studies were Identified as having a low risk of bias, indicating adequate random allocation of tissues or samples to experimental groups, while the remaining 5 studies were classified as unclear due to insufficient reporting. Baseline characteristics demonstrated high reliability, with all 9 studies identified as low risk, reflecting that tissues or samples were comparable in terms of size, type, and source. In contrast, allocation concealment, random housing, and random outcome selection were consistently identified as unclear across all 9 studies, indicating a widespread lack of information on these methodological aspects. Only one study was classed as a low risk for treatment blinding. Similarly, blinding of treatment and blinding of results were often insufficiently described. Only 3 studies were identified as low risk for blinding of results, while the remaining 6 studies were unclear. Incomplete outcome data was identified as unclear in all 9 studies, as most failed to provide adequate details on the handling of missing data. In contrast, selective outcome reporting and other sources of bias were consistently identified as low risk across all 9 studies, indicating good adherence to reporting all pre-specified outcomes and a lack of significant design-related bias.

Overall, none of the ex vivo studies were identified as having a low risk of bias across all assessed domains. The highest number of domains identified as “Low” in a single study was five. Only two studies achieved a “Low” rating in sequence generation, baseline characteristics, result blinding, selective outcome reporting, and other sources of bias domains: Cooper et al (2019) and Sandhu et al (2017). Whilst Liu et al. (2025) was identified as low risk in baseline characteristics, treatment blinding, result blinding, selective outcome reporting, and other sources of bias domains.

#### Quality assessment of in vitro studies using the SciRAP tool

The quality assessment of in vitro TKI rodent toxicity studies were evaluated using the SciRAP evaluation tool. Each study was assessed across multiple domains, including test compound and controls, test system, administration of the test compound, data collection and analysis, and funding/competing interests. Criteria were identified as"F"(fulfilled),"PF"(partially fulfilled), or"NF"(not fulfilled). The detailed results are summarised in Table 4 for assessing the reporting quality and in Table 5 for assessing the methodology quality.

**Table 4:** Assessment of reporting quality across in vitro tyrosine kinase inhibitor (TKI) rodent toxicity studies using the SciRAP evaluation tool

References	Test compound and Controls			Test system				Administration of test compound			Data collection and analysis				Funding/competing interest
	1	4	5	6	7	10	11	13	15	16	17	19	20	21	22/23
Barr et al. (2014)	F	F	F	F	F	F	F	F	F	F	F	F	F	F	F
Burke et al. (2019)	F	F	F	F	F	F	F	F	F	F	F	F	F	F	F
French et al. (2010)	F	F	F	F	F	F	NF	F	F	F	F	F	F	F	NF
Hasinoff et al. (2017)	F	F	F	F	F	F	F	F	F	F	F	F	F	F	F
Herman et al. (2011)	F	NF	F	F	F	F	F	F	F	F	F	F	F	F	F
Jacob et al. (2016)	F	F	F	F	F	F	F	F	F	F	F	F	F	F	F
Kobara et al. (2021)	F	F	F	F	F	F	F	F	F	F	F	F	F	F	F
Li et al. (2023a)	F	F	F	F	F	F	NF	F	F	F	F	F	F	F	F
Liu et al. (2023)	F	F	F	F	F	F	F	F	F	F	F	F	F	F	F
Ma et al. (2020a)	F	NF	F	F	F	NF	NF	F	F	F	F	F	F	F	F
McMullen et al. (2021)	F	F	F	F	F	F	F	F	F	F	F	F	F	F	F
Pentassuglia et al. (2009)	F	F	F	F	F	F	F	F	F	F	F	F	F	F	F
Shi et al. (2025)	F	F	F	F	F	F	F	F	F	F	F	F	F	F	F
Stuhlmiller et al. (2017)	F	F	F	F	F	F	NF	F	F	F	F	F	F	F	F
Tousif et al. (2023)	F	F	F	F	F	F	F	F	F	F	F	F	F	F	F
Wolf et al. (2010)	F	F	F	F	F	F	F	F	F	F	F	F	F	F	F
Wolf et al. (2011)	F	F	F	F	F	F	F	F	F	F	F	F	F	F	F
Xu et al. (2022)	F	F	F	F	F	F	F	F	F	F	F	F	F	F	F
Xu et al. (2024a)	F	F	F	F	F	F	F	F	F	F	F	F	F	F	F
Yang et al. (2024)	F	NF	F	F	F	F	PF	F	F	F	F	F	F	F	F

Each row corresponds to an individual study, while each column represents a specific reporting quality criterion."F"indicates that the criterion was fulfilled, “PF” indicates partially fulfilled, whereas"NF"indicates that the criterion was not fulfilled. The criteria span multiple domains, including the test compound and controls, test system, administration of the test compound, data collection and analysis, and funding/competing interests

**Table 5:** Assessment of the methodology quality of in vitro tyrosine kinase inhibitor (TKI) rodent toxicity studies using the SciRAP evaluation tool

References	Test compound and controls				Test system		Administration of test compound			Data collection and analysis			
	1	3	4	5	6	7	8	9	10	11	12	13	15
Barr et al. (2014)	F	F	F	PF	F	F	F	F	F	F	F	F	F
Burke et al. (2019)	F	F	F	NF	F	F	F	F	F	F	F	F	F
French et al. (2010)	F	F	F	F	F	F	F	F	F	F	F	F	F
Hasinoff et al. (2017)	F	F	F	NF	F	F	F	F	F	F	F	F	F
Herman et al. (2011)	F	NF	NF	NF	F	F	F	F	F	F	F	F	F
Jacob et al. (2016)	F	F	F	NF	F	F	F	F	F	F	F	F	F
Kobara et al. (2021)	F	F	F	F	F	F	F	F	F	F	F	F	F
Li et al. (2023a)	F	F	F	NF	F	F	F	F	F	F	F	F	F
Liu et al. (2023)	F	F	F	NF	F	F	F	F	F	F	F	F	F
Ma et al. (2020a)	F	NF	NF	PF	F	NF	F	F	F	F	F	F	F
McMullen et al. (2021)	F	F	F	PF	F	F	F	F	NF	F	F	F	F
Pentassuglia et al. (2009)	F	F	F	NF	F	F	F	F	F	F	F	F	F
Shi et al. (2025)	F	F	F	NF	F	F	F	F	F	F	F	F	F
Stuhlmiller et al. (2017)	F	F	F	NF	F	PF	F	F	F	F	F	F	F
Tousif et al. (2023)	F	F	F	NF	F	F	F	F	F	F	F	F	F
Wolf et al. (2010)	F	F	F	NF	F	F	F	F	F	F	F	F	F
Wolf et al. (2011)	F	F	F	PF	F	F	F	F	F	F	F	F	F
Xu et al. (2022)	F	F	F	NF	F	F	F	F	F	F	F	F	F
Xu et al. (2024a)	F	F	F	NF	F	F	F	F	F	F	F	F	F
Yang et al. (2024)	F	NF	NF	NF	F	NF	F	F	F	F	F	F	F

Each row corresponds to an individual study, while each column represents a specific reporting quality criterion."F"indicates that the criterion was fulfilled, “PF” indicates partially fulfilled, whereas"NF"indicates that the criterion was not fulfilled. The criteria span multiple domains, including the test compound and controls, test system, administration of the test compound, data collection and analysis, and funding/competing interests

#### SciRAP-reporting quality analysis

Across the test compound and controls domain, most criteria were fulfilled. All 21 studies fully reported essential details, including the chemical name of the TKI (criterion 1) and the inclusion of a vehicle or untreated control (criterion 5). However, three studies were identified as not fulfilled due to the lack of identification of the drug vehicle (criterion 4). Within the test system domain, all 21 studies adequately described the primary cell or test system (criterion 6) and the source animal from which the cell was derived (criterion 7). Despite this, one study did not report the composition of the media used (criterion 10), and five studies failed to specify incubation conditions, such as temperature, humidity, or CO_2_ concentration (criterion 11). These omissions led to their classification as not fulfilled for these criteria. Across the administration of the test compound and data collection and analysis domains (criteria 13, 15–21), all 21 studies fulfilled the required criteria, indicating strong adherence to reporting standards in these domains. In the funding and conflicts of interest domain (criteria 22 and 23), one study was classified as not fulfilled due to the absence of disclosures regarding funding sources or potential competing interests.

Among the in vitro studies assessed; 15 studies met all 15 reporting quality criteria evaluated using the SciRAP tool. The studies that achieved full compliance included Barr et al (2014), Burke et al (2019), Hasinoff et al (2017), Jacob et al (2016), Kobara et al (2021), Liu et al (2023), McMullen et al (2021), Pentassuglia et al (2009), Shi et al. (2025), Tousif et al (2023), Wolf et al (2010 and 2011) and, Xu et al (2022, 2024a, b) (Table 4).

#### SciRAP-methods quality reporting

Across the test compound and controls domain, most criteria were fulfilled. All 21 studies fully reported the name of the TKI (criterion 1). However, three studies were identified as not fulfilled due to not identifying and/or including a drug vehicle (criteria 3 and 4). Criterion 5 in the methodology quality assessment requires the use of a positive control, two studies were classified to have fulfilled this, four partially fulfilled this whilst 14 did not fulfil this criterion. Within the test system domain, all studies, met the criteria of using a sensitive and reliable test system (criterion 6). Two studies did not fulfil criterion 7, which required appropriate culturing/maintenance conditions for the cell and another study partially fulfilled this. Within the administration of test compound domain, all 21 studies met the criteria of using suitable exposure duration (criterion 8) and suitable concentrations (criterion 9). One study did not fulfil criterion 10, which requires that the test conditions during and after the drug exposure are suitable, due to lack of reporting. Across the administration of the test compound and data collection and analysis domains (criteria 11–13 and 15), all 21 studies fulfilled the required criteria, indicating strong adherence to methodological standards within data analysis. Among the assessed in vitro studies, two studies demonstrated exemplary methods of reporting by fulfilling all 13 methodological quality criteria assessed by the SciRAP tool. These studies included French et al (2010), and Kobara et al (2021) (Table 5**).**

## Discussion

**Fig. 3:**
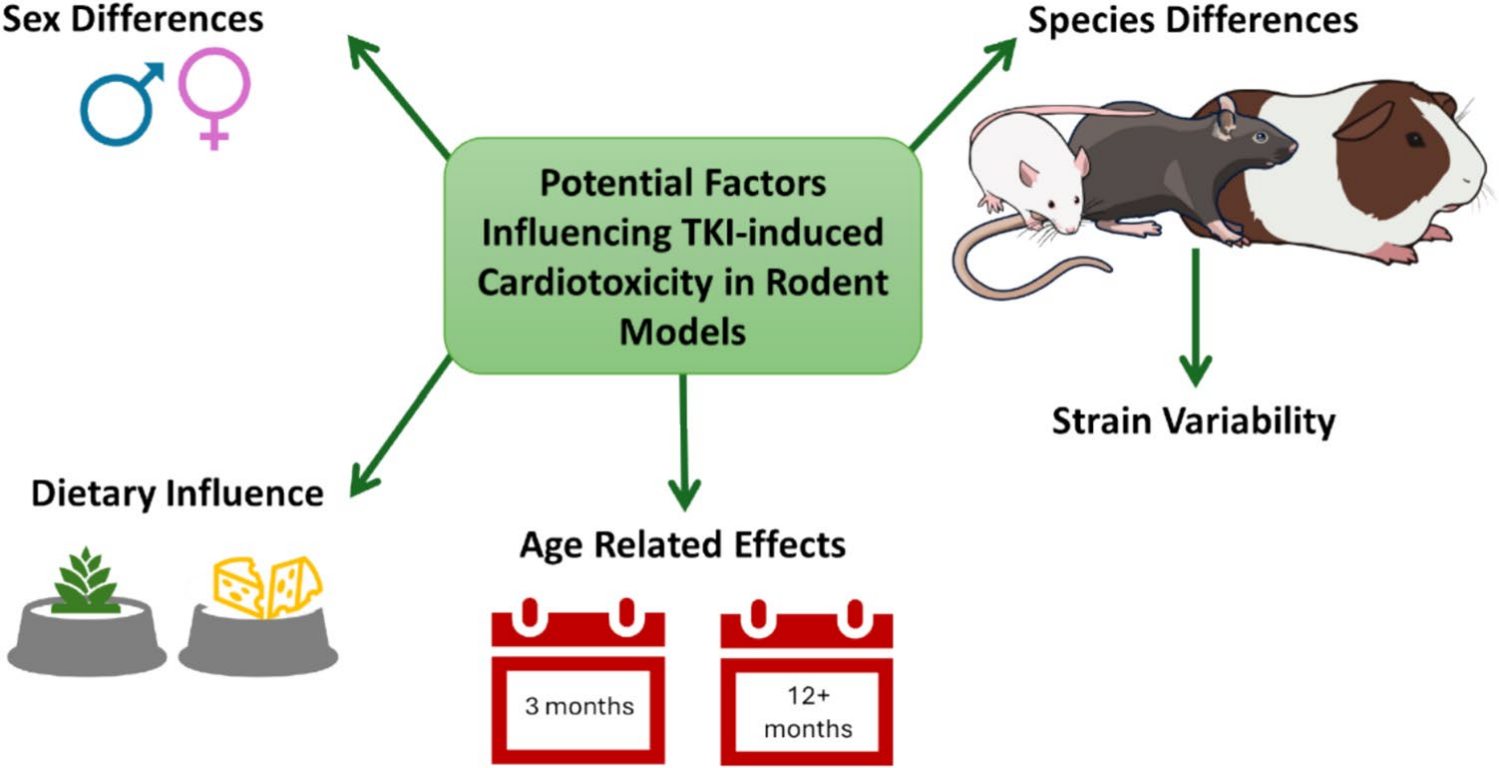
Potential factors influencing TKI-induced cardiotoxicity in rodent models. The systematic review identified several factors potentially affecting rodent cardiac responses to TKI treatment including sex, age, diet, species and strain. Image created using NIH BioArt Source [NIAID Visual & Medical Arts. (1/27/2025). Guinea Pig. NIAID NIH BIOART Source. bioart.niaid.nih.gov/bioart/578, NIAID Visual & Medical Arts. (10/7/2024). Lab Mouse. NIAID NIH BIOART Source. bioart.niaid.nih.gov/bioart/283, NIAID Visual & Medical Arts. (10/7/2024). Black Rat. NIAID NIH BIOART Source. bioart.niaid.nih.gov/bioart/54]

This systematic review provides a comprehensive assessment of TKI-induced cardiotoxicity in experimental rodent models. Our findings highlight the significant variability of studies with respect to the species of model (rats, mice, guinea pigs), strains, sex, and age ranges, as well as experimental design, range of TKI dosing regimens, and outcome measurements (Fig. 3). Methodological differences were observed in how cardiovascular parameters were measured, including echocardiographic techniques, biomarker assessments, and histopathological analyses. Many studies only reported qualitative or semi-quantitative data rather than standardised numerical data, limiting direct comparison between studies and restricting the feasibility of statistical pooling. Given these factors, a narrative synthesis was chosen to provide a structured, comprehensive interpretation of the available evidence, while accounting for the complexities and limitations of the included studies.

Our data has significant implications for both preclinical research and clinical translation. The frequent occurrence of adverse cardiac events in rodent models is consistent with clinical observations of TKI-induced cardiotoxicity in patients, including alterations in heart rate, arrhythmias, hypertension and indications of heart failure, reinforcing the relevance of these animal models in modelling of human responses (Agarwal et al. 2018; Chang et al. 2023; Chen et al. 2008). However, variation in experimental design, dosing regimens, and rodent strains, sex and age presents a significant challenge in drawing definitive conclusions that translate to advancing the understanding of the underlying pathophysiological mechanisms of human responses.

Our findings support the need to refine the selection of preclinical models to improve their predictive accuracy and strengthen comparative analyses. The identification of possible sex, age, diet, species and strain-specific differences in TKI-mediated cardiotoxic responses highlights the need for more standardised, comprehensive preclinical study design. These differences identified between studies suggest that TKI-induced cardiotoxicity is influenced by biological variables that are not adequately accounted for in current preclinical models, limiting their ability to predict patient-specific outcomes. The studies that investigated and identified sex-specific responses, and age-related susceptibility reinforced the necessity of incorporating diverse biological conditions into experimental frameworks (Cooper et al. 2019; Madonna et al. 2021, 2022; Maharsy et al. 2014).

The selection of animal species is important in cardiotoxicity studies because differences in cardiovascular physiology, electrophysiology, and drug metabolism can influence susceptibility and severity of disease. Whilst rats and mice were the commonly used models, they have significantly faster heart rates (300–500 bpm) and shorter ventricular action potential (25–50 ms) compared to humans (60–100 bpm and 250 ms) (Farraj et al. 2011). Their ECG profiles also differ, as rats and mice lack a well-defined T-wave, which makes assessing drug-induced effects on ventricular repolarisation challenging (Mitchell et al. 1998). In contrast, guinea pigs have a cardiac repolarisation profile more akin to humans, including a well-defined T-wave and action potential duration comparable to humans (Joukar 2021). This makes guinea pigs a more suitable model for studying TKI-induced electrocardiographic changes such as arrhythmias, which are key cardiotoxic effects associated with TKIs (Chang et al. 2023; Grela-Wojewoda et al. 2022). However, despite this advantage, guinea pigs were rarely used in the included studies, limiting their potential contribution to understanding human-relevant electrophysiological responses to TKIs.

Guinea pigs and other species also demonstrate distinct physiological responses to TKIs, further highlighting the importance of species selection beyond ECG comparability. Sunitinib increased heart rate in rats (Blanca et al. 2016; Blasi et al. 2012) but reduced heart rate in guinea pigs (Mooney et al. 2015). Imatinib increased left ventricular mass and wall thickness in rats (Heyen et al. 2013; Savi et al. 2018) but reduced both of these parameters in mice (Maharsy et al. 2014). While erlotinib caused elevated LVIDs in mice (Jensen et al. 2017b), in rats both LVIDs and LVIDd was decreased (Mak et al. 2015). These contrasting outcomes demonstrate how species-specific differences in drug metabolism, cardiac structure, and regulatory mechanisms markedly influence cardiotoxic profiles, and further support the value of cross-species comparisons in preclinical studies.

Differences between species strain were rarely explored, with most studies using Sprague–Dawley rats and C57Bl/6 mice without comparison to other strains. While additional strains such as Wistar, Albino Wistar, Spontaneously Hypertensive Rats (SHR), and FVB/N mice were used, only one paper evaluated strain-induced variation in TKI response (Herman et al. 2011). Herman et al (2011) identified that the inbred strain SHR experienced more adverse cardiac effects than the outbred SD strain (Herman et al. 2011). Specifically, the SHR rats were found to have higher cTnT I level and more severe cardiomyopathy than in SD rats (Herman et al. 2011). It was postulated that it was the hypertensive phenotype of the SHR rats that was responsible for this sensitivity to imatinib treatment compared to the normotensive SD rats (Herman et al. 2011). This relationship between hypertension and TKI-induced cardiotoxicity is also reported in humans and is listed as a known risk factor for cardiotoxicity (Kaddoura et al. 2023; Poku 2023). It is therefore likely that any strain of species with a predisposition to hypertension will be more sensitive to TKI-induced cardiotoxicity.

The sex of animals used was also rarely considered, with most studies using only male rodents or failing to report the sex of the animals. Very few studies included both sexes of animals or performed analyses on sex-dependent susceptibility and mediation of TKI-induced cardiotoxicity. From the studies that did identify the sex of rodents used, the data was conflicted as to which sex had a higher risk of cardiotoxicity (Harvey and Leinwand 2015; Madonna et al. 2021, 2022). In mice treated with ponatinib, female mice were found to be less susceptible to the TKI-induced cardiotoxicity (Madonna et al. 2021, 2022). However, in the case of sunitinib cardiotoxicity, female mice showed an increased risk (Harvey and Leinwand 2015). Harvey and Leinwand postulated that this increased risk was a result of endogenous levels of oestradiol, the primary form of oestrogen in mammals (Harvey and Leinwand 2015).

Considering the established differences between male and female human cardiovascular systems the lack of sex-balanced investigation is a major limitation to the understanding of TKI-mediated cardiotoxicity as male rodent-dominated studies potentially skew research data to male physiology (St Pierre et al. 2022). Compared to males, human females have an increased BPM and a reduced CO (St Pierre et al. 2022). These physiological differences may have a role in driving differential response and adverse drug reaction between males and females. Of the ten drugs withdrawn from the US market between 1997 and 2000, eight were found to pose a greater health risk to women than to men (Heinrich et al. 2001). It is noteworthy that seven of these withdrawn drugs were due to cardiovascular events (Heinrich et al. 2001). This highlights a critical research gap to be considered in pre-clinical trials, with potential harm for patients, particularly women.

Age was another poorly reported variable, with studies reporting a wide range of ages, from neonates to aged rodents and some failing to report the animal’s age. Only two studies evaluated age-related differences in susceptibility to TKI-induced cardiotoxicity with conflicting results. Cooper et al. (2019) observed that younger rats (3–12 months) were more susceptible to sunitinib-induced cardiotoxicity, whereas Maharsy et al. (2014) reported a greater risk of imatinib-induced cardiotoxicity in older mice (Cooper et al. 2019; Maharsy et al. 2014). These findings suggest species- and age-related differences in TKI cardiotoxicity, with the association of age and risk being TKI-dependent (Cooper et al. 2019; Maharsy et al. 2014). The findings of Maharsy et al., are consistent with clinical observations that older patients are at a greater risk of developing TKI-induced cardiotoxicity, particularly those over 75 years of age (Sayegh et al. 2023). Age-related changes, such as increased myocardial fibrosis, and reduced regenerative capacity, contribute to elevated susceptibility (Anwar et al. 2024; Murtha et al. 2019). Additionally, the phenomenon of inflammaging, chronic, low-grade inflammation associated with age, may exacerbate the adverse cardiac effects of TKIs (Ajoolabady et al. 2023). However, most studies within our dataset did not systematically assess age-related differences, limiting broader conclusions on how aging influences TKI-induced cardiotoxicity. Future research should incorporate age-stratified analyses to better predict long-term cardiac risks in aging populations.

The diet of the animal during or prior to the experiment may contribute to susceptibility to TKI-induced cardiotoxicity in rodents. Although Tousif et al. (2023) did not directly compare dietary effects on TKI response, they observed that ApoE−/− mice on a high-fat diet (HFD) exhibited significant reduction in EF and FS, as well as decreased LVIDs, following ponatinib treatment, whereas C57BL/6J mice on a normal chow diet showed no response (Tousif et al. 2023). As this experiment was across two different strains, ApoE^−/−^, which is prone to atherosclerosis, and C57BL/6J, which is prone to cardiac hypertrophy, strain-specific differences are likely to contribute to the observed variation in cardiotoxic response (Garcia-Menendez et al. 2013; Lo Sasso et al. 2016; Tousif et al. 2023). Positive nutritional interventions, such as the use of antioxidants, have been shown to reduce the risk of chemotherapy-induced cardiotoxicity in humans (Stephenson et al. 2024; Zhang et al. 2022). This highlights the importance of diet and nutrition in both rodents and humans in modulating susceptibility to drug-induced cardiotoxicity. Considering dietary influences in preclinical models could improve translatability to human outcomes and help identify risk-reducing or risk-increasing diets in TKI-induced cardiotoxicity.

A striking observation from our review was the dominant reliance on in vivo models alone, with 80 out of 92 studies using live rodents in experiments. Few studies used in vitro or ex vivo methods, and far fewer combined different approaches, with only 14 study including in vivo and in vitro experiments, two studies combining in vivo and ex vivo and one using all three experimental groups. Whilst in vitro and ex vivo models are limited in their ability to measure the complex physiological systems observed in in vivo models, we propose that their under representation in these studies represents a missed opportunity to improve the quality and depth of data by delineating the biological mechanisms that underpin the outcomes of in vivo experiments. Further benefit can be derived by using in vitro and ex vivo models to inform experimental design to reduce the number of animals used and refine experiments to reduce the severity of harm to the animal. In vivo studies can involve more animal suffering, especially when testing TKIs, which are known to cause harmful side effects. Administering these drugs to live animals and observing them over time may lead to considerable distress. Although combining model types may not reduce animal use directly, it could improve the quality and depth of the data.

The risk of bias analysis conducted using SYRCLE’s tool for in vivo and ex vivo studies and SciRAP for in vitro studies revealed several methodological shortcomings (Hooijmans et al. 2014; Roth et al. 2021). One of the most significant concerns was the lack of transparency in randomisation procedures and allocation concealment. Many studies failed to describe how experimental groups were assigned, leading to an unclear risk of selection bias. This issue was particularly prominent in in vivo studies, where over half of the included research did not provide sufficient details on sequence generation.

Blinding of investigators and outcome assessors was also inconsistently applied, raising concerns about detection and performance biases. Only 20 in vivo studies explicitly stated that blinding was used during outcome assessment, meaning that in most cases, assessors may have been aware of treatment allocation, potentially influencing data interpretation. Similarly, in ex vivo studies, only three studies ensured proper blinding to result assessors, further increasing the likelihood of bias in outcome measurements. The absence of adequate blinding in many studies limits confidence in the objectivity of reported results.

The assessment of in vitro studies using the SciRAP tool further highlighted inconsistencies in experimental design and reporting. Several studies failed to specify the exact drug vehicle, necessary for distinguishing TKI-induced effects from potential solvent-related artifacts. Most studies also failed to include a positive control, which would reveal if the chosen model was sensitive enough for the experiment. Several studies also had incomplete reporting of cell maintenance conditions which would affect the reproducibility of the experiment. Only two studies demonstrated strong methodological rigor, whilst many exhibited gaps in essential reporting criteria, limiting their reproducibility and reliability. The findings of the risk of bias analysis emphasise the need for greater methodological standardisation, improved reporting practices, and enhanced transparency in preclinical rodent TKI-induced cardiotoxicity studies.

In conclusion, this systematic review highlights the widespread occurrence of TKI-induced cardiotoxicity in rodent models, whilst underscoring the need for improved experimental design and reporting to enhance translational relevance. The variability in cardiotoxicity parameters is influenced by multiple factors, including species, strain, diet, sex, age, and experimental conditions. While rodent models effectively replicate several key aspects of human TKI cardiotoxicity, limitations such as the predominant use of male rodents, a lack of strain comparisons, and poor consideration of age-related differences constrain their predictive accuracy. The limited use of guinea pigs, despite their electrophysiological similarities to humans, further represents a missed opportunity in preclinical research. Additionally, the risk of bias analysis revealed significant methodological shortcomings, particularly regarding randomisation, blinding, and reporting of experimental details, which reduce the reliability and reproducibility of findings. Addressing these limitations through standardised study designs, sex-balanced cohorts, age-stratified analyses, and improved methodological rigor will be crucial in refining preclinical cardiotoxicity models. Future research should focus on integrating these factors to generate more robust and clinically translatable data, ultimately improving the translatability in human patients.

## Supplementary Information

Below is the link to the electronic supplementary material.

Supplementary file1 (DOCX 136 KB)

Supplementary file2 (DOCX 25 KB)

Supplementary file3 (DOCX 18 KB)

Supplementary file4 (DOCX 17 KB)

Supplementary file5 (DOCX 17 KB)

Supplementary file6 (DOCX 18 KB)

Supplementary file7 (DOCX 18 KB)

Supplementary file8 (DOCX 21 KB)

Supplementary file9 (DOCX 18 KB)

Supplementary file10 (DOCX 47 KB)

Supplementary file11 (DOCX 53 KB)

Supplementary file12 (DOCX 49 KB)

Supplementary file13 (DOCX 23 KB)

Supplementary file14 (DOCX 32 KB)

Supplementary file15 (DOCX 35 KB)

Supplementary file16 (DOCX 35 KB)

Supplementary file17 (DOCX 25 KB)

Supplementary file18 (DOCX 46 KB)

Supplementary file19 (DOCX 72 KB)

Supplementary file20 (DOCX 27 KB)

## Data Availability

All data generated or analysed during this study are included in this published article and its supplementary information files.
