# Supplementary material for: Evaluating the use of rodents as in vitro, in vivo and ex vivo experimental models for the assessment of tyrosine kinase inhibitor-induced cardiotoxicity: a systematic review
Source: Arch Toxicol. 2025 Sep 11;99(12):4801–28. doi: 10.1007/s00204-025-04159-0 (PMC12534346; doi:10.1007/s00204-025-04159-0)
Supplement: Supplementary file 1 — Supplementary file1 (DOCX 136 KB) [file 204_2025_4159_MOESM1_ESM.docx]

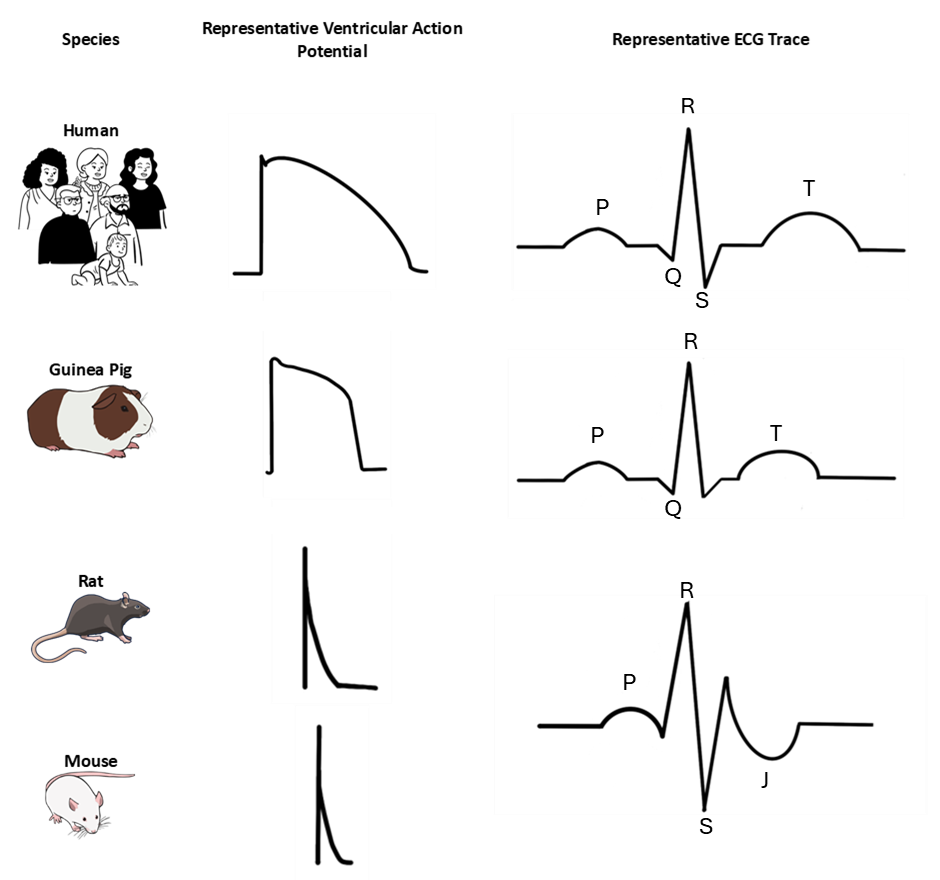


Supplemental Figure 1 Species differences in ventricular action potential and ECG trace between humans, guinea pigs, rats, and mice. Humans and guinea pigs show longer ventricular action potentials with a clear plateau phase and broader ECG T waves. Rats and mice exhibit short, triangular action potentials and with biphasic T waves (known as J waves) and absent Q waves. Adapted from Joukar (2021). https://creativecommons.org/licenses/by/4.0/
