# Supplementary material for: Evaluating the use of rodents as in vitro, in vivo and ex vivo experimental models for the assessment of tyrosine kinase inhibitor-induced cardiotoxicity: a systematic review
Source: Arch Toxicol. 2025 Sep 11;99(12):4801–28. doi: 10.1007/s00204-025-04159-0 (PMC12534346; doi:10.1007/s00204-025-04159-0)
Supplement: Supplementary file 3 — Supplementary file3 (DOCX 18 KB) [file 204_2025_4159_MOESM3_ESM.docx]

**Supplemental Table 2 Population, Interventions, Comparisons, Outcomes, Timing and Setting (PICOTS) Framework for the Systematic Review on Cardiotoxicity of TKIs in Rodent Models.** The PICOTS framework outlines the structure of the systematic review, including Population (rodent models such as rats, mice, hamsters, gerbils, and guinea pigs), Intervention (exposure to tyrosine kinase inhibitors), Comparator (vehicle control or no treatment control), Outcomes (cardiotoxicity parameters, including physiological measures, histopathological changes, biomarker levels, and animal clinical reports), Timing (all exposure times studied), and Setting (*in vivo*, *in vitro*, and *ex vivo* experimental models).

| **PICOTS Element** | **Details** |
| --- | --- |
| Population | Rodents (rats, mice, hamsters, gerbils and guinea pigs) used in cardiotoxicity testing of tyrosine kinase inhibitors (TKI). |
| Intervention | TKI treatment.  TKI’s include; Acalabrutinib, Afatinib, Avapritinib, Axitinib, Bosutinib, Brigatinib, Cabozantinib, Crizotinib, Dabrafenib, Dasatinib, Dacomitinib, Erlotinib, Ensartinib, Fedratinib, Gilteritinib, Idelalisib, Imatinib, Ibrutinib, Lapatinib, Larotrectinib, Lenvatinib, Midostaurin, Nilotinib, Osimertinib, Pazopanib, Pexidartinib, Ponatinib, Quizartinib, Regorafenib, Ripretinib, Ruxolitinib, Sorafenib, Sunitinib, Trametinib, Vandetanib, Zanubrutinib, Umbralisib.  Unlicensed/non-marketed TKI drugs can also be included so long as they have reported TKI activity. |
| Comparison | Comparison of study designs; *in vitro*, *ex vivo* and *in vivo*  Comparison of rodent model characteristics; species, strain, age, sex and numbers used  Comparison of cardiac adverse event outcomes: including physiological parameters, histopathological parameters, animal clinical report  Comparison of tyrosine kinase inhibitors used within studies; name, dose and exposure. |
| Outcome | **Primary Outcomes**  Cardiac adverse event outcomes: including physiological parameters, histopathological parameters, animal clinical report.  **Secondary Outcomes**  Animal Clinical Reports (Cardiac related)   - Mortality/Survival Rates - Reports of arrhythmia - Reports of hypertension - Reports of myocardial infarction - Reports of heart failure   Histopathological Changes   - Reports of fibrosis - Reports of inflammation/cellular infiltration - Reports of necrosis - Other changes   Significant Physiological Changes in   - Cardiac Output - Stroke Volume - Heart Rate - Ejection Fraction - Fractional Shortening - Left Ventricular Wall Thickness - Left Ventricular Mass - Left Ventricular Internal Dimension - Ejection Time - QT Interval - PR Interval - QRS Duration   Significant Biomarker Outcomes   - Troponin Levels - Myoglobin Levels - Creatine Kinase-MB (CK-MB) Levels - B-type Natriuretic Peptide (BNP) Levels |
| Timing | Studies of all durations will be included in the search in order to evaluate TKI induced cardiotoxicity in *in vivo*, *ex vivo*, and primary *in vitro* rodent models. |
| Setting | Laboratory setting |
