# Supplementary material for: Evaluating the use of rodents as in vitro, in vivo and ex vivo experimental models for the assessment of tyrosine kinase inhibitor-induced cardiotoxicity: a systematic review
Source: Arch Toxicol. 2025 Sep 11;99(12):4801–28. doi: 10.1007/s00204-025-04159-0 (PMC12534346; doi:10.1007/s00204-025-04159-0)
Supplement: Supplementary file 4 — Supplementary file4 (DOCX 17 KB) [file 204_2025_4159_MOESM4_ESM.docx]

**Supplemental Table 3 Boolean Search Strategy for Identifying Studies Relevant to TKI-Induced Cardiotoxicity in Rodent Models Across Bibliographic Databases.** A Boolean search strategy was applied across bibliographic databases, PubMed, Web of Science, and Scopus, to identify studies investigating tyrosine kinase inhibitor-induced cardiotoxicity in rodent models. The search terms incorporated Boolean operators (AND, OR) to combine concepts related to rodent species, TKI treatments, and cardiotoxic outcomes. Truncation symbols (*) and wildcards facilitated the retrieval of relevant variations of key terms across different indexing systems.

| **Search Strategy** | **Details** |
| --- | --- |
| Databases | Pubmed  Web of Science  Scopus |
| Search terms for Pubmed | ("rodent" OR "mouse" OR "Mus" OR "rats" OR “rat” OR "Rattus" OR "meriones" OR "mesocricetus" OR "cavia" OR "hamster" OR “hamsters” OR "gerbil" OR “gerbils” OR "guinea pig" OR “guinea pig”) AND ("tyrosine kinase inhibition" OR "TKI" OR "acalabrutinib" OR "afatinib" OR "avapritinib" OR "axitinib" OR "bosutinib" OR "brigatinib" OR "cabozantinib" OR "crizotinib" OR "dabrafenib" OR "dasatinib" OR "dacomitinib" OR "erlotinib" OR "ensartinib" OR "fedratinib" OR "gilteritinib" OR "idelalisib" OR "imatinib" OR "ibrutinib" OR "lapatinib" OR "larotrectinib" OR "lenvatinib" OR "midostaurin" OR "nilotinib" OR "osimertinib" OR "pazopanib" OR "pexidartinib" OR "ponatinib" OR "quizartinib" OR "regorafenib" OR "ripretinib" OR "ruxolitinib" OR "sorafenib" OR "sunitinib" OR "trametinib" OR "vandetanib" OR "zanubrutinib" OR "umbralisib") AND ("cardiotoxic" OR "cardiotoxicity" OR "cardiac toxicity" OR "heart toxicity" OR "cardiac adverse effects" OR "cardiac adverse events" OR "arrhythmia" OR "cardiomyopathy" OR "valvulopathy" OR "myocardial infarction" OR "myocardial dysfunction" OR "heart failure" OR "MI" OR "left ventricular dysfunction" OR "electrocardiogram" OR "electrocardiography" OR "ECG" OR "EKG" OR "echocardiography" OR "echocardiogram" OR "electrophysiology" OR "cardiac arrest" OR “cardiac event” OR "cardiovascular safety") |
| Search terms for Web of Science | ("rodent" OR "mouse" OR “mice” OR "Mus" OR "rat" OR “rats” OR "rattus" OR "meriones" OR "mesocricetus" OR "cavia" OR "hamster*" OR "gerbil*" OR "guinea pig*") AND ("tyrosine kinase inhibit*" OR "TKI") AND ("cardiotox*" OR "cardiac toxicity" OR "heart toxicity" OR "cardiac adverse*" OR "arrhythmia" OR "cardiomyopathy" OR "valvulopathy" OR "myocardial*" OR "heart failure" OR "MI" OR "left ventricular dysfunction" OR "electrocardiogram" OR “ECG” OR “EKG” OR "echocardio*" OR “electrophysiology” OR “cardiovascular safety” OR “cardiac arrest” OR “cardiac event”) |
| Search terms for Scopus | ("rodent" OR "mouse" OR “mice” OR "Mus" OR "rat" OR “rats” OR "rattus*" OR "meriones" OR "mesocricetus" OR "cavia" OR "hamster*" OR "gerbil*" OR "guinea pig*") AND ("tyrosine kinase inhibit*" OR "TKI" OR "*tinib" OR "dabrafenib" OR "idelalisib" OR "midostaurin" OR "pazopanib" OR "regorafenib" OR "sorafenib" OR "umbralisib") AND ("cardiotox*" OR "cardiac toxicity" OR "heart toxicity" OR "cardiac adverse*" OR "arrhythmia" OR "cardiomyopathy" OR "valvulopathy" OR "myocardial*" OR "heart failure" OR "MI" OR "left ventricular dysfunction" OR "electrocardio*" OR “ECG” OR “EKG” OR "echocardio*" OR “electrophysiology” OR “cardiac event” OR “cardiovascular safety” OR “cardiac event”) |
