# Supplementary material for: Evaluating the use of rodents as in vitro, in vivo and ex vivo experimental models for the assessment of tyrosine kinase inhibitor-induced cardiotoxicity: a systematic review
Source: Arch Toxicol. 2025 Sep 11;99(12):4801–28. doi: 10.1007/s00204-025-04159-0 (PMC12534346; doi:10.1007/s00204-025-04159-0)
Supplement: Supplementary file 5 — Supplementary file5 (DOCX 17 KB) [file 204_2025_4159_MOESM5_ESM.docx]

**Supplemental Table 4 Inclusion and Exclusion Criteria Applied to Studies.** Inclusion and exclusion criteria were established to determine the eligibility of studies for the systematic review. Studies were considered eligible if they were primary research articles published in English and focused on the cardiotoxic effects of tyrosine kinase inhibitors (TKIs) in rodent models. Eligible studies included, *in vivo*, *ex vivo*, and primary *in vitro* experiments using rats, mice, hamsters, guinea pigs, or gerbils, with a primary focus on cardiac adverse event outcomes such as physiological, histopathological, biomarker, and clinical parameters. Studies were considered regardless of publication year or methodological quality, provided they met the inclusion criteria.

| **Inclusion Criteria** | **Exclusion Criteria** |
| --- | --- |
| Primary research articles | Abstract, Conference proceedings, systematic reviews, reviews and case reports |
| English language | Non-English language material |
| Rodents; Rats, mice, hamsters, guinea pigs, and gerbils  Model: primary *in vitro*, *ex vivo* and *in vivo* | *In vivo*, *ex vivo* and *in vitro* studies on non-rodents, and *in vitro* studies using rodent immortalised cells |
| Rodent primary cell/tissue/animal exposure to TKI | Cell/tissue/animal not exposed to TKI |
| Cardiac adverse event outcomes, including physiological parameters, histopathological parameters, animal clinical report  and biomarker levels post-TKI exposure | Study does not assess listed cardiac adverse event outcomes post TKI exposure |
| Controlled studies with separate control group (vehicle control/ no treatment control) | Case studies, cross-over studies, studies without a separate control group. |
