# Supplementary material for: Evaluating the use of rodents as in vitro, in vivo and ex vivo experimental models for the assessment of tyrosine kinase inhibitor-induced cardiotoxicity: a systematic review
Source: Arch Toxicol. 2025 Sep 11;99(12):4801–28. doi: 10.1007/s00204-025-04159-0 (PMC12534346; doi:10.1007/s00204-025-04159-0)
Supplement: Supplementary file 6 — Supplementary file6 (DOCX 18 KB) [file 204_2025_4159_MOESM6_ESM.docx]

**Supplemental Table 5 Signalling Questions for the SYRCLE’s Tool for Assessing Risk of Bias for Animal Studies (*In Vivo*).** The SYRCLE’s tool was used to assess the risk of bias of *in vivo* rodent TKI-induced cardiotoxicity studies. The tool was also adapted for the use in *ex vivo* studies. Each study was critically appraised across different sources of bias, including selection, performance, detection, attrition, and reporting bias. Within these sources of bias, specific signalling questions were used to assess specific domains such as sequence generation, baseline characteristics, allocation concealment, random housing, blinding (treatment), random outcome selection, blinding (results), incomplete outcome data, selective outcome reporting and other sources of bias. The signalling questions from the SYRCLE’s tool was also adapted to suit *ex vivo* studies. Studies were also evaluated for the presence of additional sources of bias that could influence study validity. This systematic approach ensured a comprehensive assessment of potential biases affecting the reliability of reported findings.

| **Type of Bias** | **Domain** | **SYRCLE’s Signalling Questions for *In vivo* Studies** | **Adapted SYRCLE’s Signalling Questions for Ev-vivo Studies** |
| --- | --- | --- | --- |
| Selection Bias | Sequence generation | Was the allocation sequence adequately generated and applied? | Was the allocation of tissues or samples to experimental groups random and adequately described? |
|  | Baseline characteristics | Were the groups similar at baseline or were they adjusted for confounders in the analysis? | Were the tissues or samples comparable in terms of characteristics such as size, type, and source? |
|  | Allocation Concealment | Was the allocation adequately concealed? | Was treatment allocation blinded? |
| Performance Bias | Random Housing | Were the animals randomly housed during the experiment? | Were the animals where the tissue was derived from housed randomly |
|  | Blinding (Treatment) | Were the caregivers and /or investigators blinded from knowledge which intervention each animal received during the experiment? | Were the researchers analysing the samples blinded to the treatment groups? |
| Detection Bias | Random Outcome Selection | Were animals selected at random for outcome assessment? | Was the order of outcome assessments randomized? |
|  | Blinding (Results) | Was the outcome assessor blinded? | Were the researchers analysing the outcome data blinded? |
| Attrition Bias | Incomplete Outcome Data | Were incomplete outcome data adequately addressed? | Was there any missing data or samples and have the paper described how missing data was handled? |
| Reporting Bias | Selective Outcome Reporting | Are reports of the study free of selective outcome reporting? | Have all that all pre-specified outcomes are reported? Report any additional outcomes measured transparently. |
| Other | Other Sources of Bias | Was the study apparently free of other problems that could result in high risk of bias? | Is the study design free from other source of bias? |
