# Supplementary material for: Evaluating the use of rodents as in vitro, in vivo and ex vivo experimental models for the assessment of tyrosine kinase inhibitor-induced cardiotoxicity: a systematic review
Source: Arch Toxicol. 2025 Sep 11;99(12):4801–28. doi: 10.1007/s00204-025-04159-0 (PMC12534346; doi:10.1007/s00204-025-04159-0)
Supplement: Supplementary file 7 — Supplementary file7 (DOCX 18 KB) [file 204_2025_4159_MOESM7_ESM.docx]

**Supplemental Table 6 Adaptation of the SciRAP Tool for Reporting Quality Assessment**. The assessment of reporting quality was conducted using an adapted version of the SCIRAP (Science in Risk Assessment and Policy) tool to evaluate the completeness and transparency reported *in vitro* studies. As per SciRAP recommendations, criteria deemed irrelevant to the study were removed. Each *in vitro* study was evaluated using this adapted tool. The evaluation covered key reporting categories, including test compound and controls, test system, administration of the test compound, data collection and analysis, and funding and competing interests. Each category contained specific criteria assessing whether essential details, such as chemical identification, test system description, dosing parameters, experimental conditions, analytical methods, statistical reporting, and disclosure of funding and competing interests, were clearly documented.

| **Domain** | **SCIRAP Numbered Criteria** | **Criteria** |
| --- | --- | --- |
| Test Compound and Controls | 1 | The chemical name or other identification, such as CAS-number, of the test compound was given. |
|  | 4 | The solvent (vehicle) was described. |
|  | 5 | It was stated that a solvent (vehicle) or no treatment control was included. |
| Test System | 6 | The test system (e.g., cell line/cells/tissue/organ/embryo/sub-cellular fractions) was described. |
|  | 7 | The source of the test system was stated. |
|  | 10 | Composition of media was described, including the use of serum, antibiotics, etc. |
|  | 11 | Incubation temperature, humidity, and CO_2_ concentration were described. |
| Administration of Test Compound | 13 | The administered dose levels or concentrations were stated. |
|  | 15 | The duration of treatment was stated. |
|  | 16 | The number of replicates per dose level/concentration or the number of times the experiment was repeated was stated. |
| Data Collection and Analysis | 17 | The tests and/or analytical methods used were sufficiently described to allow for evaluation of the reliability of results. |
|  | 19 | It was stated that the effect of the test compound on cytotoxicity was measured. |
|  | 20 | All results were clearly presented. |
|  | 21 | The statistical methods and software used were described. |
| Funding and Competing Interests | 22 | The funding sources for the study were stated. |
|  | 23 | Any competing interests were disclosed, or it was explicitly stated that the authors did not have any competing interests. |
