# Supplementary material for: Evaluating the use of rodents as in vitro, in vivo and ex vivo experimental models for the assessment of tyrosine kinase inhibitor-induced cardiotoxicity: a systematic review
Source: Arch Toxicol. 2025 Sep 11;99(12):4801–28. doi: 10.1007/s00204-025-04159-0 (PMC12534346; doi:10.1007/s00204-025-04159-0)
Supplement: Supplementary file 8 — Supplementary file8 (DOCX 21 KB) [file 204_2025_4159_MOESM8_ESM.docx]

**Supplemental Table 7 Adaptation of the SciRAP Tool for Methodology Quality Assessment.** The assessment of methodological quality was conducted using an adapted version of the SciRAP tool to evaluate the rigor and reliability of experimental procedures. Each *in vitro* study was evaluated using this adapted tool. The evaluation covered key methodological domains, including test compound and controls, test system, administration of the test compound, and data collection and analysis. Each category included specific criteria to ensure methodological soundness, such as appropriate chemical identification, solvent and control selection, suitability of test systems, exposure conditions, reliability of analytical methods, replication adequacy, and statistical transparency.

| **Category** | **SCIRAP Numbered Criteria** | **Criterion** |
| --- | --- | --- |
| Test Compound and Controls | 1 | The chemical name or other identification, such as CAS-number, of the test compound was given. |
|  | 3 | An appropriate solvent (vehicle) was used that is not expected to interfere with the results of the study at the concentration used. |
|  | 4 | A solvent (vehicle) control was included. |
|  | 5 | An appropriate positive control was included, and the expected result was observed from this treatment. |
| Test System | 6 | A reliable and sensitive test system (e.g., cell line/cells/tissue/organ/embryo/sub-cellular fractions) with metabolic competence, if relevant, was used for investigating the test compound and endpoints. |
|  | 7 | Conditions for cultivation and/or maintenance of the cell line/cells/tissue/organ/embryo/sub-cellular fractions (incubation temperature, humidity, CO2 concentration, media used, number of cell passages, control of contamination) were appropriate. |
| Administration of the Test Compound | 8 | The duration of exposure was suitable for the test system and investigated endpoints. |
|  | 9 | The concentrations used were suitable for the test system and investigated endpoints. |
|  | 10 | The test conditions during and after exposure to the test compound were suitable (media and serum used, cell density, incubation temperature, humidity, CO2 concentration). |
| Data Collection and Analysis | 11 | Reliable and sensitive tests and/or analytical methods were used for investigating the endpoints. |
|  | 12 | Sufficient numbers of replicates or repetitions of the experiment were used to generate reliable and valid results. |
|  | 13 | Measurements were collected at suitable time points to generate sensitive, valid, and reliable data. |
|  | 15 | The statistical methods were clearly described and do not seem inappropriate, unusual, or unfamiliar. |
