# Supplementary material for: Evaluating the use of rodents as in vitro, in vivo and ex vivo experimental models for the assessment of tyrosine kinase inhibitor-induced cardiotoxicity: a systematic review
Source: Arch Toxicol. 2025 Sep 11;99(12):4801–28. doi: 10.1007/s00204-025-04159-0 (PMC12534346; doi:10.1007/s00204-025-04159-0)
Supplement: Supplementary file 9 — Supplementary file9 (DOCX 18 KB) [file 204_2025_4159_MOESM9_ESM.docx]

**Supplemental Table 8 Rationale for exclusion of studies in full text screening.** Studies were excluded if they did not meet one or more of the predefined inclusion criteria regarding study design, model system, exposure, or outcome relevance. Sixteen studies were excluded during full text screening.

| **Reference** | **Exclusion Criteria** |
| --- | --- |
| Aharonovitz et al. 1998 | Cell/tissue/animal not exposed to TKI |
| Wu et al. 2025 |  |
| Feng et al. 2025 |  |
| Wang et al. 2025 | Study does not assess listed cardiac adverse event outcomes post TKI exposure |
| Fujii et al. 2024 | *In vivo*, *ex vivo* and *in vitro* studies on non-rodents, and *in vitro* studies using rodent immortalised cells |
| Bouitbir et al. 2022 |  |
| Fortini et al. 2024 |  |
| Alhazzani et al. 2023 | Study does not assess listed cardiac adverse event outcomes post-TKI exposure |
| Brown et al. 2005 |  |
| Cui et al. 2013 |  |
| Hasinoff et al. 2010 |  |
| Hasinoff et al. 2020 |  |
| Hasinoff et al. 2008 |  |
| Hasinoff et al. 2013 |  |
| Hasinoff et al. 2011 |  |
| Zitron et al. 2008 |  |
| Wang et al. 2023 |  |
| Schneider et al. 2018 |  |
| Qian et al. 2010 |  |
| Lu et al. 2024 |  |
| Hu et al. 2012 |  |
