# Supplementary material for: Evaluating the use of rodents as in vitro, in vivo and ex vivo experimental models for the assessment of tyrosine kinase inhibitor-induced cardiotoxicity: a systematic review
Source: Arch Toxicol. 2025 Sep 11;99(12):4801–28. doi: 10.1007/s00204-025-04159-0 (PMC12534346; doi:10.1007/s00204-025-04159-0)
Supplement: Supplementary file 10 — Supplementary file10 (DOCX 47 KB) [file 204_2025_4159_MOESM10_ESM.docx]

**Supplemental Table 9 Effect of TKIs on Heart Rate Across Rodent Models.** A summary of the effect of TKIs on heart rate (HR) across rodent models. The table includes the reference, experimental animal model, specific TKI studied, administered dose (mg/kg unless otherwise stated), duration of treatment, and observed changes in HR. Arrows and coloured cells indicate a significant increase (↑ red) or decrease (↓ blue) in HR, while "NS" denotes no significant change. "NR" represents data not reported.

| **Reference** | **Experimental Animal Model** | **TKI Studied** | **Dose (mg/kg, unless otherwise stated)** | **Exposure** | **HR- heart rate (bpm)** |
| --- | --- | --- | --- | --- | --- |
| Abdelgalil et al. 2020 | Rat | Sorafenib | 20 | 4 Weeks | ↓ |
| French et al. 2010 | Rat | Sorafenib | 10 | 3 weeks | ↓ |
| Aguirre et al. 2010 | Rat | PF-04254644 | 500 | 1-week single dose. Measured at 6 then 24 h | ↑ 6 h and ↑ 24 h |
|  |  |  | 80 |  | ↑ |
| Blanca et al. 2016 | Rat | Sunitinib | 25 | 8 weeks | ↑ |
| Blasi et al. 2012 | Rat | Sunitinib | 10 | 4 weeks daily, 2 weeks off-treatment 2 weeks on treatment | ↑ on day 31 (off-treatment) |
|  |  |  |  |  | ↓ on day 46 |
| Mooney et al. 2015 | Guinea Pig | Sunitinib | 16 | 6 days | ↓ |
| Sandhu et al. 2017 | Rat | Sunitinib | 1 µM | 125 min | ↓ |
| Cooper et al. 2019 | Rat | Sunitinib | 1 µM | 125 min | ↓3- and 12-month-old |
| Cheng et al. 2023 | Mouse | Crizotinib | 40 | 4 weeks | ↑ |
| Li et al. 2024a | Mouse | Sunitinib | 10 | 2 weeks | ↑ |
|  |  |  | 20 |  | ↓ |
|  |  |  | 40 |  | ↓ |
| Jie et al. 2021 | Guinea Pig | Gefitinib | 10 | NR | ↓ |
|  |  |  | 30 |  | ↓ |
| Henderson et al. 2013 | Rat | Sunitinib | 1-10 µM | 100 min | ↓ |
|  |  | Sorafenib | 6-10 µM |  | ↓ |
| Liu et al. 2025 | Mouse | Alectinib | 10 | 1 week | ↓ |
| Akman et al. 2014 | Rat | Pazopanib | 100 | 1 hr | NS |
| Stuhlmiller et al. 2017 | Mouse | Erlotinib | 2 weeks | 50 mg/kg | NS |
|  |  | Sunitinib |  | 40 mg/kg | NS |
|  |  | Sorafenib |  | 30 mg/kg | NS |
| Aguirre et al. 2010 | Rat | PF-04254644 | 40 | 7 Day repeat dose | NS |
|  |  |  | 80 |  | NS |
|  |  |  | 320 then 160 |  | NS |
|  |  |  | 40 | 6 Day repeat dose | NS |
| Blasi et al. 2012 | Rat | Sunitinib | 1 | 4 weeks daily, 2 weeks off-treatment 2 weeks on treatment | NS |
| French et al. 2010 | Rat | Sunitinib | 10 | 3 weeks | NS |
| French et al. 2010 | Rat | Pazopanib | 300 | 3 weeks | NS |
| Ren et al. 2021 | Mouse | Sunitinib | 40 | 4 weeks | NS |
| Qin et al. 2024 | Mouse | Sunitinib | 40 | 4 weeks | NS |
| Maharsy et al. 2014 | Mouse | Imatinib | 200 | 5 weeks | NS |
| Li et al. 2023 | Guinea Pig | Osimertinib | 0.8 | NR | NS |
|  |  |  | 2.4 |  | NS |
|  |  |  | 7.2 |  | NS |
|  |  |  | 24 |  | NS |
| Kuburas et al. 2022 | Rat | Sunitinib | 1 µM | 175 min | NS |
| Jiang et al. 2019 | Mouse | Ibrutinib | 25 | 14 weeks | NS |
| Jensen et al. 2017b | Mouse | Sunitinib | 40 | 2 weeks | NS |
|  |  | Erlotinib | 50 |  | NS |
| Jensen et al. 2017a | Mouse | Sorafenib | 30 | 2 weeks | NS |
| Henderson et al. 2013 | Rat | Sunitinib | 0.001 - 0.1 µM | 100 min | NS |
|  |  | Sorafenib | 0.1-3 µM |  | NS |
|  |  | Erlotinib | 0.001-10 µM |  | NS |
| Cooper et al. 2019 | Mice | Sunitinib | 1 µM | 125 min | NS in 24-month-old |
| Savi et al. 2018 | Rat | Imatinib | 50 | 3 weeks | NS |
|  |  |  | 100 |  | NS |
| Mozolevska et al. 2019 | Mouse | Bevacizumab | 10 | 4 weeks | NS |
|  |  | Sunitinib | 40 |  | NS |
| Bordun et al. 2015 | Mouse | Bevacizumab | 10 | 2 weeks | NS |
|  |  | Sunitinib | 40 |  | NS |
| Monogiou Belik et al. 2024 | Mouse | Quizartinib | 10 | 4 weeks | NS |
| Xu et al. 2024 | Mouse | Regorafenib | 200 | 6 weeks | NS |
| Liu et al. 2025 | Mouse | Alectinib | 1-5 µM | 15 minutes | NS |
