# Supplementary material for: Evaluating the use of rodents as in vitro, in vivo and ex vivo experimental models for the assessment of tyrosine kinase inhibitor-induced cardiotoxicity: a systematic review
Source: Arch Toxicol. 2025 Sep 11;99(12):4801–28. doi: 10.1007/s00204-025-04159-0 (PMC12534346; doi:10.1007/s00204-025-04159-0)
Supplement: Supplementary file 12 — Supplementary file12 (DOCX 49 KB) [file 204_2025_4159_MOESM12_ESM.docx]

**Supplemental Table 11** **Effect of TKIs on Fractional Shortening Across Rodent Models.** A summary of the effect of TKIs on fractional shortening (FS) across rodent models. The table includes the reference, rodent model, specific TKI studied, administered dose (mg/kg), duration of treatment, and observed changes in FS. Arrows and coloured cells indicate a significant increase (↑ red) or decrease (↓ blue) in HR, while "NS" denotes no significant change. "NR" represents data not reported.

| Reference | Experimental Animal Model | TKI Studied | Dose (mg/kg) | Duration of Treatment | Fractional Shortening (FS) |
| --- | --- | --- | --- | --- | --- |
| Kobara et al. 2021 | Mouse | Imatinib | 200 | 5 weeks | ↓ |
| Kerkelä et al. 2006 | Mouse | Imatinib | 200 | 5 weeks | ↓ |
| Maharsy et al. 2014 | Mouse | Imatinib | 200 | 5 weeks | ↓ old |
| Jensen et al. 2017a | Mouse | Sorafenib | 30 | 2 weeks | ↓ |
| Li et al. 2022 | Mouse | Sorafenib | 30 | 2 weeks | ↓ |
| Duran et al. 2014 | Mouse | Sorafenib | 30 and 40 with induced MI or sham | 3 weeks | ↓ with MI and sham |
| Stuhlmiller et al. 2017 | Mouse | Sorafenib | 30 | 2 weeks | ↓ |
| Li et al. 2024c | Mouse | Sorafenib | 30 | 2 weeks | ↓ |
| Mohamad et al. 2024 | Rat | Sunitinib | 25 | 4 weeks | ↓ |
| Li et al. 2023a | Mouse | Sunitinib | 40 | 2 weeks | ↓ |
| Qin et al. 2024 | Mouse | Sunitinib | 40 | 4 weeks | ↓ |
| Ren et al. 2021 | Mouse | Sunitinib | 40 | 4 weeks | ↓ |
| Sourdon et al. 2021 | Mouse | Sunitinib | 50 | 6 weeks | ↑ |
| Stuhlmiller et al. 2017 | Mouse | Sunitinib | 40 | 2 weeks | ↓ |
| Yang et al. 2019a | Mouse | Sunitinib | 40 | 4 weeks | ↓ |
| Yang et al. 2019b | Mouse | Sunitinib | 40 | 4 weeks | ↓ |
| Tousif et al. 2023 | Mouse | Ponatinib | 15 | 2 weeks | ↓for high fat diet |
| Madonna et al. 2021 | Mouse | Ponatinib | 30 | 4 weeks | ↓ for male and female |
| Mak et al. 2015 | Rat | Erlotinib | 10 | 9 weeks | ↓ |
| Xu et al. 2024a | Mice | Crizotinib | 100 | 6 weeks | ↓ |
| Xu et al. 2024b | Mouse | Regorafenib | 200 | 6 weeks | ↓ |
| Yang et al. 2024 | Mouse | Osimertinib | 25-50 | 3 weeks | ↓ |
| Li et al. 2024b | Rat | Ibrutinib | 30 | 4 weeks | ↓ |
| Krüger et al. 2025 | Mouse | Lenvatinib | 4 | 4 days | ↓ |
| Tousif et al. 2023 | Mouse | Ponatinib | 15 | 2 weeks | NS for chow diet |
| Stuhlmiller et al. 2017 | Mouse | Erlotinib | 50 | 2 weeks | NS |
| Shuai et al. 2023 | Rat | Ibrutinib | 25 | 4 weeks | NS |
| Xiao et al. 2020 | Mouse | Ibrutinib | 25 | 4 weeks | NS |
| Liu et al. 2023 | Rat | Sorafenib | 50 | 4 weeks | NS |
| Jensen et al. 2017b | Mouse | Sunitinib | 40 | 2 weeks | NS |
|  |  | Erlotinib | 50 |  | NS |
| Jiang et al. 2019 | Mouse | Ibrutinib | 25 | 14 weeks | NS |
| Mooney et al. 2015 | Guinea Pig | Sunitinib | 16 | 6 days | NS |
| Harvey and Leinwand 2015 | Mouse | Sunitinib | 40 | 4 weeks | NS |
| Cheng et al. 2023 | Mouse | Crizotinib | 40 | 4 weeks | NS |
| Blasi et al. 2012 | Rat | Sunitinib | 10 | 4 weeks daily, 2 weeks off-treatment 2 weeks on treatment | NS |
| Monogiou Belik et al. 2024 | Mouse | Quizartinib | 10 | 4 weeks | NS |
