# Supplementary material for: Evaluating the use of rodents as in vitro, in vivo and ex vivo experimental models for the assessment of tyrosine kinase inhibitor-induced cardiotoxicity: a systematic review
Source: Arch Toxicol. 2025 Sep 11;99(12):4801–28. doi: 10.1007/s00204-025-04159-0 (PMC12534346; doi:10.1007/s00204-025-04159-0)
Supplement: Supplementary file 13 — Supplementary file13 (DOCX 23 KB) [file 204_2025_4159_MOESM13_ESM.docx]

**Supplemental Table 12 Effect of TKIs on Stroke Volume Across Rodent Models.** Changes in stroke volume (SV) in response to TKIs across rodent models. The dataset includes reference information, species, specific TKI studied, administered dose (mg/kg), duration of treatment, and observed changes in SV. Arrows and coloured cells indicate a significant increase (↑ red) or decrease (↓ blue), while "NS" denotes no significant change and "NR" represents data not reported.

| **Reference** | **Experimental Animal Model** | **TKI Studied** | **Dose (mg/kg)** | **Duration of Treatment** | **Stroke Volume (SV)** |
| --- | --- | --- | --- | --- | --- |
| Aguirre et al. 2010 | Rat | PF-04254644 | 320/160 | 7 Day repeat dose | ↑ |
|  |  |  | 500 | 7 Day single dose. Measured at 6 then 24 h | ↑ Day 6 |
| Heyen et al. 2013 | Rat | Imatinib | 50 | 8 weeks | ↑ |
|  |  | Imatinib | 50 | 6 months | ↑ |
| Wolf et al. 2011 | Rat | Nilotinib | 40 | 4 weeks | ↑ |
|  |  |  | 80 |  | ↑ |
| Latifi et al. 2019 | Mouse | Ponatinib | 30 | 1 week | ↓ |
| Li et al. 2022 | Mouse | Sorafenib | 30 | 2 weeks | ↓ |
| Liu et al. 2023 | Rat | Sorafenib | 50 | 4 weeks | ↓ |
| Blasi et al. 2012 | Rat | Sunitinib | 1 | 4 weeks daily, 2 weeks off-treatment 2 weeks on treatment | NS |
|  |  |  | 10 |  | NS |
| Heyen et al. 2013 | Rat | Bosutinib | 50 | 8 weeks | NS |
|  |  | Bosutinib | 50 | 6 months | NS |
| Latifi et al. 2019 | Mouse | Dasatinib | 20 | 1 week | NS |
| Mak et al. 2015 | Rat | Erlotinib | 10 | 9 weeks | NS |
| Aguirre et al. 2010 | Rat | PF-04254644 | 40 | 7 Day repeat dose | NS |
|  |  |  | 80 | 7 Day repeat dose | NS |
|  |  |  | 40 | 6 Day repeat dose | NS |
|  |  |  | 80 |  | NS |
| French et al. 2010 | Rat | Sorafenib | 10 | 3 weeks | NS |
|  |  | Sunitinib | 10 |  | NS |
|  |  | Pazopanib | 300 |  | NS |
