# Supplementary material for: Evaluating the use of rodents as in vitro, in vivo and ex vivo experimental models for the assessment of tyrosine kinase inhibitor-induced cardiotoxicity: a systematic review
Source: Arch Toxicol. 2025 Sep 11;99(12):4801–28. doi: 10.1007/s00204-025-04159-0 (PMC12534346; doi:10.1007/s00204-025-04159-0)
Supplement: Supplementary file 14 — Supplementary file14 (DOCX 32 KB) [file 204_2025_4159_MOESM14_ESM.docx]

**Supplemental Table 13 Effect of TKIs on Cardiac Output Across Rodent Models.** Changes to cardiac output (CO) following TKI treatment in rodent models. Information includes the reference, species, specific TKI, administered dose (mg/kg), duration of treatment, and observed changes in CO. Arrows and coloured cells indicate a significant increase (↑ red) or decrease (↓ blue), while "NS" denotes no significant change and "NR" represents data not reported.

| **Reference** | **Experimental Animal Model** | **TKI Studied** | **Dose (mg/kg)** | **Duration of Treatment** | **Cardiac Output (CO)** |
| --- | --- | --- | --- | --- | --- |
| Aguirre et al. 2010 | Rat | PF-04254644 | 500 | 7 Day single dose. Measured at 6 then 24 h | ↑ Day 6 and 12 |
|  |  |  | 80 | 6 Day repeat dose | ↑ |
| Mak et al. 2015 | Rat | Erlotinib | 10 | 9 weeks | ↓ |
| Li et al. 2023a | Mouse | Sunitinib | 40 | 2 weeks | ↓ |
| Yang et al. 2024 | Mouse | Osimertinib | 25-50 | 3 weeks | ↓ |
| Aguirre et al. 2010 | Rat | PF-04254644 | 40 | 7 Day repeat dose | NS |
|  |  |  | 80 |  | NS |
|  |  |  | 320/160 |  | NS |
|  |  |  | 40 | 6 Day repeat dose | NS |
| Blasi et al. 2012 | Rat | Sunitinib | 1 | 4 weeks daily, 2 weeks off-treatment 2 weeks on treatment | NS |
|  |  |  | 10 |  | NS |
| French et al. 2010 | Rat | Sorafenib | 10 | 3 weeks | NS |
|  |  | Sunitinib | 10 |  | NS |
|  |  | Pazopanib | 300 |  | NS |
