# Supplementary material for: Evaluating the use of rodents as in vitro, in vivo and ex vivo experimental models for the assessment of tyrosine kinase inhibitor-induced cardiotoxicity: a systematic review
Source: Arch Toxicol. 2025 Sep 11;99(12):4801–28. doi: 10.1007/s00204-025-04159-0 (PMC12534346; doi:10.1007/s00204-025-04159-0)
Supplement: Supplementary file 15 — Supplementary file15 (DOCX 35 KB) [file 204_2025_4159_MOESM15_ESM.docx]

Supplemental Table 14 Effect of TKIs on Left Ventricular Mass Across Rodent Models. Changes to left ventricular mass induced by TKI treatment in rodent models. The summary includes reference details, species, specific TKI, administered dose (mg/kg), duration of treatment, and observed changes in left ventricular mass. Arrows and coloured cells indicate a significant increase (↑ red) or decrease (↓ blue), while "NS" denotes no significant change and "NR" represents data not reported.

| **Reference** | **Experimental Animal Model** | **TKI Studied** | **Dose (mg/kg)** | **Duration of**  **Treatment** | **Left Ventricular Mass (LVM)** |
| --- | --- | --- | --- | --- | --- |
| Heyen et al. 2013 | Rat | Imatinib | 50 | 8 weeks | ↑ |
| Jensen et al. 2017a | Mouse | Sorafenib | 30 | 2 weeks | ↑ |
| Sourdon et al. 2021 | Mouse | Sunitinib | 50 | 6 weeks | ↑ |
| Harvey and Leinwand 2015 | Mouse | Sunitinib | 40 | 4 weeks | ↓ female |
| Maharsy et al. 2014 | Mouse | Imatinib | 200 | 5 weeks | ↓ Young |
| Jiang et al. 2019 | Mouse | Ibrutinib | 25 | 14 weeks | NS |
| Savi et al. 2018 | Rat | Imatinib | 50 | 3 weeks | NS |
|  |  |  | 100 |  | NS |
| Wolf et al. 2011 | Rat | Nilotinib | 40 | 4 weeks | NS |
|  |  |  | 80 |  | NS |
| Heyen et al. 2013 | Rat | Bosutinib | 50 | 8 weeks | NS |
|  |  |  |  | 6 months | NS |
|  |  | Imatinib |  |  | NS |
| Harvey and Leinwand 2015 | Mouse | Sunitinib | 40 | 4 weeks | NS male |
| Stuhlmiller et al. 2017 | Mouse | Erlotinib | 59 | 2 weeks | NS |
|  |  | Sunitinib | 40 |  | NS |
|  |  | Sorafenib | 30 |  | NS |
| Maharsy et al. 2014 | Mouse | Imatinib | 200 | 5 weeks | NS old |
