# Supplementary material for: Evaluating the use of rodents as in vitro, in vivo and ex vivo experimental models for the assessment of tyrosine kinase inhibitor-induced cardiotoxicity: a systematic review
Source: Arch Toxicol. 2025 Sep 11;99(12):4801–28. doi: 10.1007/s00204-025-04159-0 (PMC12534346; doi:10.1007/s00204-025-04159-0)
Supplement: Supplementary file 16 — Supplementary file16 (DOCX 35 KB) [file 204_2025_4159_MOESM16_ESM.docx]

Supplemental Table 15 Effect of TKIs on Left Ventricular Wall Thickness Across Rodent Models. A summary of the effect of TKI treatment on left ventricular wall thickness across different rodent models. The table includes the reference, rodent model, specific TKI studied, administered dose (mg/kg), duration of treatment, and observed changes in left ventricular wall thickness. Arrows and coloured cells indicate a significant increase (↑ red) or decrease (↓ blue) in HR, while "NS" denotes no significant change. "NR" represents data not reported.

| **Reference** | **Experimental Animal Model** | **TKI Studied** | **Dose (mg/kg)** | **Duration of Treatment** | **Left Ventricular Thickness** |
| --- | --- | --- | --- | --- | --- |
| Harvey and Leinwand 2015 | Mouse | Sunitinib | 40 | 4 weeks | ↓ female |
| Maharsy et al. 2014 | Mouse | Imatinib | 200 | 5 weeks | ↓ young and old |
| Mak et al. 2015 | Rat | Erlotinib | 10 | 9 weeks | ↓ |
| Ren et al. 2021 | Mouse | Sunitinib | 40 | 4 weeks | ↑ |
| Savi et al. 2018 | Rat | Imatinib | 50 | 3 weeks | ↑ |
|  |  |  | 100 |  | ↑ |
| Wolf et al. 2011 | Rat | Nilotinib | 40 | 4 weeks | ↑ |
|  |  |  | 80 |  | ↑ |
| Heyen et al. 2013 | Rat | Imatinib | 50 | 8 weeks | ↑ |
|  |  |  |  | 6 months | ↑ |
| Harvey and Leinwand 2015 | Mouse | Sunitinib | 40 | 28 days | NS male |
| Wolf et al. 2010 | Rat | Imatinib | 200 | 5 weeks | NS |
| Mozolevska et al. 2019 | Mouse | Bevacizumab | 10 | 4 weeks | NS |
|  |  | Sunitinib | 40 |  | NS |
| Jiang et al. 2019 | Mouse | Ibrutinib | 25 | 14 weeks | NS |
| Heyen et al. 2013 | Rat | Bosutinib | 50 | 8 weeks | NS |
|  |  | Bosutinib |  | 6 months | NS |
