# Supplementary material for: Evaluating the use of rodents as in vitro, in vivo and ex vivo experimental models for the assessment of tyrosine kinase inhibitor-induced cardiotoxicity: a systematic review
Source: Arch Toxicol. 2025 Sep 11;99(12):4801–28. doi: 10.1007/s00204-025-04159-0 (PMC12534346; doi:10.1007/s00204-025-04159-0)
Supplement: Supplementary file 17 — Supplementary file17 (DOCX 25 KB) [file 204_2025_4159_MOESM17_ESM.docx]

Supplemental Table 16 Effect of TKIs on Left Ventricular Internal Dimension at Diastole and Systole Across Rodent Models. Left ventricular internal dimension at diastole (LVIDd) and systole (LVIDs) following TKI treatment in rodent models. The summary provides reference details, species, specific TKI, administered dose (mg/kg), duration of treatment, and observed changes in LVIDd and LVIDs. Arrows and coloured cells indicate a significant increase (↑ red) or decrease (↓ blue), while "NS" denotes no significant change and "NR" represents data not reported.

| **Reference** | **Experimental Animal Model** | **TKI Studied** | **Dose (mg/kg)** | **Duration of Treatment** | **Left Ventricular Internal Dimension at diastole (LVIDd)** | **Left Ventricular Internal Dimension at systole (LVIDs)** |
| --- | --- | --- | --- | --- | --- | --- |
| Mozolevska et al. 2019 | Mouse | Bevacizumab | 10 | 4 weeks | ↑ | NR |
|  |  | Sunitinib | 40 |  | ↑ | NR |
| Maharsy et al. 2014 | Mouse | Imatinib | 200 | 5 weeks | ↑ Old | NR |
| Tousif et al. 2023 | Mouse | Ponatinib | 15 | 2 weeks | ↑ for high fat diet | NR |
| Mattii et al. 2024 | Mouse | Ponatinib | 30 | 4 weeks | ↑ | ↑ |
| Krüger et al. 2025 | Mouse | Lenvatinib | 4 | 4 days | ↑ | ↑ |
| Kerkelä et al. 2006 | Mouse | Imatinib | 200 | 5 weeks | ↓ | ↓ |
| Mak et al. 2015 | Rat | Erlotinib | 10 | 9 weeks | ↓ | ↓ |
| Liu et al. 2023 | Rat | Sorafenib | 50 | 4 weeks | NR | ↓ |
| Jensen et al. 2017b | Mouse | Sunitinib | 40 | 2 weeks | NS | ↑ |
|  |  | Erlotinib | 50 |  | NS | ↑ |
| Ren et al. 2021 | Mouse | Sunitinib | 40 | 4 weeks | NS | ↑ |
| Stuhlmiller et al. 2017 | Mouse | Sunitinib | 40 | 2 weeks | NS | ↑ |
| Maharsy et al. 2014 | Mouse | Imatinib | 200 | 5 weeks | NS Young | NR |
| Yang et al. 2024 | Mouse | Osimertinib | 25-50 | 3 weeks | NS | NR |
| Monogiou Belik et al. 2024 | Mouse | Quizartinib | 10 | 4 weeks | NS | NS |
| Madonna et al. 2021 | Mouse | Ponatinib | 30 | 4 weeks | NS | NS |
| Jensen et al. 2017a | Mouse | Sorafenib | 30 | 2 weeks | NS | NS |
| Jiang et al. 2019 | Mouse | Ibrutinib | 25 | 14 weeks | NS | NS |
| Shuai et al. 2023 | Rat | Ibrutinib | 25 | 4 weeks | NS | NS |
| Stuhlmiller et al. 2017 | Mouse | Erlotinib | 50 | 2 weeks | NS | NS |
|  |  | Sorafenib | 30 |  | NS | NS |
| Li et al. 2024b | Rat | Ibrutinib | 30 | 4 weeks | NS | NS |
| Xiao et al. 2020 | Mouse | Ibrutinib | 25 | 4 weeks | NS | NR |
| Yan et al. 2024 | Rat | Ibrutinib | 17 | 4 weeks | NS | NS |
| Tousif et al. 2023 | Mouse | Ponatinib | 15 | 2 weeks | NS | NR |
| Cheng et al. 202 | Mouse | Crizotinib | 40 | 4 weeks | NS | NS |
