# Supplementary material for: Evaluating the use of rodents as in vitro, in vivo and ex vivo experimental models for the assessment of tyrosine kinase inhibitor-induced cardiotoxicity: a systematic review
Source: Arch Toxicol. 2025 Sep 11;99(12):4801–28. doi: 10.1007/s00204-025-04159-0 (PMC12534346; doi:10.1007/s00204-025-04159-0)
Supplement: Supplementary file 18 — Supplementary file18 (DOCX 46 KB) [file 204_2025_4159_MOESM18_ESM.docx]

Supplemental Table 17 Elevated Levels of Troponin in Response to TKI Treatment in Rodents. Troponin, a key biomarker of cardiac injury, was measured in rodent models following exposure to TKIs. The dataset includes reference information, species, specific TKI studied, administered dose (mg/kg), duration of treatment, and observed changes in troponin levels. Arrows and coloured cells indicate a significant increase (↑ red) or no significant change (NS white). Several studies reported elevated troponin levels in response to TKI treatment.

| **Reference** | **Experimental Animal Model** | **TKI Studied** | **Dose (mg/kg, unless otherwise stated)** | **Duration of Treatment** | **Troponin** |
| --- | --- | --- | --- | --- | --- |
| Alanazi et al. 2022 | Rat | Gefitinib | 30 | 3 weeks | ↑ |
| AlAsmari et al. 2020 | Rat | Gefitinib | 30 | 3 weeks | ↑ |
| Alhoshani et al. 2020 | Rat | Gefitinib | 30 | 2 weeks | ↑ |
| Aldemir et al. 2020 | Rat | Sunitinib | 25 | 5 weeks | ↑ |
| Bouitbir et al. 2019 | Mouse | Sunitinib | 7.5 | 2 weeks | ↑ |
| Qin et al. 2024 | Mouse | Sunitinib | 40 | 4 weeks | ↑ |
| Xu et al. 2022 | Mouse | Sunitinib | 40 | 32 days | ↑ |
| Ren et al. 2021 | Mouse | Sunitinib | 40 | 4 weeks | ↑ |
| Bordun et al. 2015 | Mouse | Sunitinib | 40 | 2 weeks | ↑ |
| Herman et al., 2014 | Rat | Imatinib | 200 | 4 weeks | ↑ |
| Abdel-Wahab et al. 2025 | Rat | Imatinib | 40 | 4 weeks | ↑ |
| Madonna et al. 2021 | Mouse | Ponatinib | 30 | 4 weeks | ↑ male and female |
| Cosgun et al. 2021 | Rat | Vandetanib | 25 | 1 month | ↑ |
| Bordun et al. 2015 | Mouse | Bevacizumab | 10 | 15 days | ↑ |
| Yang et al. 2024 | Mouse | Osimertinib | 25-50 | 3 weeks | ↑ |
| Herman et al. 2014 | Rat | Imatinib | 50 | 4 weeks | NS |
|  |  |  | 100 |  | NS |
|  |  |  | 100 |  | NS |
| Li et al. 2022 | Rat | Sorafenib | 50 | 14 weeks | NS |
|  |  |  |  | 28 weeks | NS |
| Aguirre et al. 2010 | Rat | PF-04254644 | 40 | 7 Day repeat dose | NS |
|  |  |  | 80 |  | NS |
|  |  |  | 320/160 |  | NS |
|  |  |  | 500 | 7 Day single dose | NS |
|  |  |  | 40 | 6 Day repeat dose | NS |
|  |  |  | 80 |  | NS |
| Wolf et al. 2011 | Rat | Nilotinib | 40 | 4 weeks | NS |
|  |  |  | 80 |  | NS |
| Mooney et al. 2015 | Guinea Pig | Sunitinib | 16 | 6 days | NS |
|  |  | Imatinib | 18 | 6 days | NS |
| Herman et al. 2011 | Rat | Imatinib | 10 | 10 days | NS |
|  |  |  | 30 |  | NS |
|  |  |  | 50 |  | NS |
|  |  |  | 50 | 2 weeks | NS |
|  |  |  | 100 |  | NS |
|  |  |  | 100 |  | NS |
| Henderson et al. 2013 | Rat | Sorafenib | 0.1-10 µM | 20 min | NS |
|  |  | Sunitinib | 0.001-0.01 µM |  | NS |
|  |  | Erlotinib | 0.001-10 µM |  | NS |
