# Supplementary material for: Evaluating the use of rodents as in vitro, in vivo and ex vivo experimental models for the assessment of tyrosine kinase inhibitor-induced cardiotoxicity: a systematic review
Source: Arch Toxicol. 2025 Sep 11;99(12):4801–28. doi: 10.1007/s00204-025-04159-0 (PMC12534346; doi:10.1007/s00204-025-04159-0)
Supplement: Supplementary file 19 — Supplementary file19 (DOCX 72 KB) [file 204_2025_4159_MOESM19_ESM.docx]

**Supplemental Table 18 Creatine Kinase and Creatine Kinase-MB Response to TKI treatment in Rodents.** Creatine kinase (CK) and CK-MB, markers of cardiac injury, were measured in rodent models following treatment with TKIs. The dataset includes reference information, species, specific TKI studied, administered dose (mg/kg), duration of treatment, and observed changes in CK or CK-MB levels. Arrows and coloured cells indicate a significant increase (↑ red), or a significant decrease (↓ blue), or both a significant increase and decrease was reported (↑ ↓pink), while "NS" denotes no significant change in CK or CK-MB and “NR” denotes it was unreported in the study.

| **Reference** | **Experimental Animal Model** | **TKI Studied** | **Dose (mg/kg, unless otherwise stated)** | **Duration of Treatment** | **CK** | **CK-MB** |
| --- | --- | --- | --- | --- | --- | --- |
| Alanazi et al. 2022 | Rat | Gefitinib | 30 | 3 weeks | NR | ↑ |
| AlAsmari et al. 2020 | Rat | Gefitinib | 30 | 3 weeks | NR | ↑ |
| Bouitbir et al. 2019 | Mouse | Sunitinib | 7.5 | 2 weeks | ↑ | NR |
| Cosgun et al. 2021 | Rat | Vandetanib | 25 | 1 month | NR | ↑ |
| Imam et al. 2020 | Rat | Sunitinib | 25 | 3 weeks | NR | ↑ |
| Mohamad et al. 2024 | Rat | Sunitinib | 25 | 4 weeks | NR | ↑ |
| Li et al. 2022 | Mouse | Sorafenib | 30 | 2 weeks | NR | ↑ |
| Maayah et al. 2014 | Rat | Sunitinib | 100 | 1 month | ↑ | ↑ |
| Patyna et al. 2008 | Rat | Sunitinib | 6 | 6 months | ↑ | NR |
| Qin et al. 2024 | Mouse | Sunitinib | 40 | 4 weeks | ↑ | NR |
| Sayed-Ahmed et al. 2019 | Rat | Sunitinib | 25 | 4 weeks | NR | ↑ |
| Abdel-Wahab et al. 2025 | Rat | Imatinib | 40 | 4 weeks | NR | ↑ |
| Song et al. 2022 | Mouse | Imatinib | 50 | 2 weeks | ↑ | NR |
|  |  |  | 100 |  | ↑ | NR |
| Xu et al. 2024 | Mouse | Crizotinib | 100 | 6 weeks | NR | ↑ |
| Xu et al. 2022 | Mouse | Sunitinib | 40 | 32 days | NR | ↑ |
| Song et al. 2023 | Mouse | Imatinib | 50 | 2 weeks | ↑ | NR |
| Korashy et al. 2016 | Rat | Gefitinib | 30 | 3 weeks | ↑ | ↑ |
|  |  |  | 20 |  | NR | ↑ |
| Yang et al. 2024 | Mouse | Osimertinib | 25-50 | 3 weeks | NR | ↑ |
| Jacob et al. 2016 | Rat | Erlotinib | 1-150 µM | 24 h and 48 h | NR | ↑↓ |
|  |  | Gefitinib |  |  | NR | ↑ |
|  |  | Lapatinib |  |  | NR | ↑↓ |
|  |  | Sunitinib |  |  | NR | ↑ |
|  |  | Imatinib |  |  | NR | ↑↓ |
|  |  | Sorafenib |  |  | NR | ↑↓ |
|  |  | Vandetanib |  |  | NR | ↑ |
|  |  | Lestaurtinib |  |  | NR | ↑ |
|  |  | Dasatinib |  |  | NR | ↓ |
| Liu et al. 2023 | Rat | Sorafenib | 50 | 14 weeks | ↓ | NR |
| Song et al. 2022 | Mouse | Imatinib | 25 | 2 weeks | NS | NR |
| Liu et al. 2023 | Rat | Sorafenib | 50 | 28 weeks | NS | NR |
| Korashy et al. 2016 | Rat | Gefitinib | 20 | 3 weeks | NS | NR |
| Mao et al. 2012 | Rat | SIM010603 | 5 | 4 weeks | NS | NR |
|  |  |  | 10 |  | NS | NR |
|  |  |  | 20 |  | NS | NR |
|  |  | Sunitinib | 10 |  | NS | NR |
| Lim et al. 2010 | Mouse | Sunitinib | 25 | 4 h | NS | NR |
|  |  |  | 50 |  | NS | NR |
|  |  |  | 80 |  | NS | NR |
|  |  |  | 100 |  | NS | NR |
|  |  |  | 140 |  | NS | NR |
| Wolf et al. 2011 | Rat | Nilotinib | 40 | 4 weeks | NS | NR |
|  |  |  | 80 |  | NS | NR |
