# Supplementary material for: Evaluating the use of rodents as in vitro, in vivo and ex vivo experimental models for the assessment of tyrosine kinase inhibitor-induced cardiotoxicity: a systematic review
Source: Arch Toxicol. 2025 Sep 11;99(12):4801–28. doi: 10.1007/s00204-025-04159-0 (PMC12534346; doi:10.1007/s00204-025-04159-0)
Supplement: Supplementary file 20 — Supplementary file20 (DOCX 27 KB) [file 204_2025_4159_MOESM20_ESM.docx]

**Supplemental Table 19 Elevated Brain Natriuretic Peptide (BNP) in Response to TKI Treatment in Rodents.** BNP and its precursor, NT-proBNP, are key biomarkers of cardiac injury, and were measured in rodent models following treatment with TKIs. The dataset includes reference information, species, specific TKI studied, administered dose (mg/kg), duration of treatment, and observed changes in troponin levels. Arrows indicate a significant increase (↑), or no significant change (NS) and “NR” denotes it was unreported in the study. Several studies reported elevated BNP or NT-proBNP in response to TKI treatment.

| **Reference** | **Experimental Animal Model** | **TKI Studied** | **Dose (mg/kg, unless otherwise stated)** | **Duration of Treatment** | **BNP** | **NT-proBNP** |
| --- | --- | --- | --- | --- | --- | --- |
| Alanazi et al. 2022 | Rat | Gefitinib | 30 | 3 weeks | NR | ↑ |
| AlAsmari et al. 2020 | Rat | Gefitinib | 30 | 3 weeks | NR | ↑ |
| Korashy et al. 2016 | Rat | Gefitinib | 30 | 3 weeks | ↑ | NR |
| Imam et al. 2020 | Rat | Sunitinib | 25 | 3 weeks | ↑ | NR |
| Ren et al. 2021 | Mouse | Sunitinib | 40 | 4 weeks | NR | ↑ |
| Maayah et al. 2014 | Rat | Sunitinib | 100 | 1 month | ↑ | NR |
| Li et al. 2022 | Mouse | Sorafenib | 30 | 2 weeks | ↑ | NR |
| Harvey and Leinwand 2015 | Rat NRVMs | Sunitinib | 150 ng/ml | 36 h | ↑ | NR |
| Harvey and Leinwand 2015 | Rat ARVMs | Sunitinib | 150 ng/ml | 36 h | ↑ female | NR |
| Korashy et al. 2016 | Rat | Gefitinib | 20 | 3 weeks | NS | NR |
| Harvey and Leinwand 2015 | Rat ARVMs | Sunitinib | 150 ng/ml | 36 h | NS male | NR |
